# Dynamic active-site induced by host-guest interactions boost the Fenton-like reaction for organic wastewater treatment

**DOI:** 10.1038/s41467-023-39228-4

**Published:** 2023-06-15

**Authors:** Dongpeng Zhang, Yanxiao Li, Pengfei Wang, Jinyong Qu, Yi Li, Sihui Zhan

**Affiliations:** 1 Key Laboratory of Pollution Processes and Environmental Criteria (Ministry of Education), College of Environmental Science and Engineering, Nankai University, Tianjin, China; 2 Tianjin Key Lab Clean Energy & Pollutant Control, School of Energy and Environmental Engineering, Hebei University of Technology, 300130 Tianjin, China; 3 Tianjin Key Laboratory of Molecular Optoelectronic Sciences, Department of Chemistry, School of Science, Tianjin University & Collaborative Innovation Center of Chemical Science and Engineering (Tianjin), 300072 Tianjin, China; 4 Joint School of National University of Singapore and Tianjin University, Fuzhou International Campus, Tianjin University, Binhai New City, 350207 Fuzhou, China

**Keywords:** Pollution remediation, Heterogeneous catalysis, Pollution remediation

## Abstract

In heterogeneous catalysis, uncovering the dynamic evolution of active sites in the working conditions is crucial to realizing increased activity and enhanced stability of catalyst in Fenton-like activation. Herein, we capture the dynamic changes in the unit cell of Co/La-SrTiO_3_ catalyst during the exemplary peroxymonosulfate activation process using X-ray absorption spectroscopy and in situ Raman spectroscopy, revealing the substrate tuned its structural evolution, which is the reversible stretching vibration of O-Sr-O and Co/Ti-O bonds in different orientations. This process effectively promotes the generation of key SO_5_* intermediates, which is beneficial to the formation of ^1^O_2_ and SO_4_^•−^ from persulfate on the Co active site. Density functional theory and X-ray absorption spectroscopy show that the optimized structural distortion enhanced the metal-oxygen bond strength by tuning the *e*_g_ orbitals and increased the number of transferred electrons to peroxymonosulfate by about 3-fold, achieving excellent efficiency and stability in removing organic pollutants.

## Abstract (web summary)

Uncovering the dynamic evolution of active sites in the working conditions is crucial to realizing increased activity and enhanced stability of catalyst in Fenton-like activation. Herein, the authors capture the dynamic changes in the unit cell of Co/La-SrTiO3 catalyst during the exemplary peroxymonosulfate activation process.

## Introduction

Faced with excessive fossil fuel consumption as well as the associated environmental issues, the implementation of carbon neutrality to control global warming in the Paris Climate Agreement is urgently demanded^1,2^. As an indispensable basic resource and an important environmental carrier, the importance of water resources has been increasingly demonstrated. At present, the discharge of non-biodegradable organic pollutants has caused severe water pollution, and Fenton-like process based on persulfates (i.e, peroxydisulfate (PDS) and peroxymonosulfate (PMS)) have received extensive attention in water treatment^3,4^. The initial step for persulfates activation is to achieve electron transfer through the adsorption of persulfates on the metal center^5^, and then generate reactive species, including radicals (i.e., O_2_^•−^, •OH, and SO_4_^•−^)^6^ and nonradical species (i.e.,^1^O_2_, high-valence metals and metals–PMS complex)^7,8^. In recent years, based on the regulation of the microenvironment of metal active sites, substantial efforts have been made to explore metal active sites with higher electron transfer rates and understand the mechanisms of free radicals conversion in persulfates activation^9,10^. However, in the actual reaction process, due to the interface hybridization between the catalyst and the reactant molecules, the local structure of the catalyst may also change dynamically^11–13^.

Actually, recent work has shown that the chemical activity and stability of the metal-based catalysts are highly dependent on the local coordination of the metal site, which is directly related to the interaction between the electron and the geometric structure^14–16^. For example, the reversible shrinkage of the M–M’ and M(M’)–O bonds in the oxide is beneficial for the increase of the electron intensity on the active site, thereby promoting the adsorption and activation of H_2_O molecules during the hydrogen evolution reaction (HER) reaction^17^. The combination of oxo-containing intermediate species (i.e., O*, OH*, OOH*) in the metal center may cause the contraction of the bond length between the metal and its coordination atom, thereby enhancing the stability of the metal atom during oxygen evolution reaction (OER)^18^. Substrate strain-tuned geometric deformation of the active site can achieve strong M–O interactions and maintain a balance between bonding to the original surrounding atoms^19^. Moreover, the host-guest van der Waals interactions in the straight channels of the zeolite framework were confirmed to lead to the deformation of the nanopore structure during the adsorption–desorption process^20^. This interaction between the host catalyst and the guest molecule can lead to a better understanding of the complex microscopic mechanism behind the macroscopic behavior of adsorption–desorption^21^. In this case, dynamic structures can significantly affect the electron transfer and contacts between the host catalyst and guest molecule, which can be exploited as a powerful tool to design novel functional catalysts. Therefore, elucidating the dynamic changes of the structure during the reaction is of great significance for better understanding the origin of catalyst catalytic activity and revealing the complex heterogeneous catalytic mechanism^22^. However, the dynamic evolutions of the catalysts have not been aware of in Fenton-like activation for a long time. More importantly, quite limited optimization strategies have been proposed so far to regulate the structural change process, and the relationship between the dynamic structure and the catalytic activity is still unclear. In view of the perovskite structure (ABO_3_) has great flexibility in regulating both electronic and surface structure at the same time, we reasoned that it could be a promising candidate for studying dynamic evolutions and stable active-site regulation in interfacial reactions.

In this work, we designed the electronic and surface structural modifications in SrTiO_3_ unit cell via La and Co doping (STLC) to realize its dynamic changes. The relationship between the dynamic changes of the unit cell in STLC and its catalytic performance in Fenton-like reaction was investigated for the first time. The results implied that PMS and *o*-nitrophenol (ONP) molecules can be effectively adsorbed on the STLC and induce the reversible stretching vibration of O–Sr–O and Co/Ti–O bonds in different orientations during the reaction. X-ray absorption spectroscopy (XAS) combined with density functional theory (DFT) calculations indicated that the regular dynamic structural change of STLC enhanced the metal-oxygen bond strength by tuning the *e*_g_ orbitals, facilitating the electron transfer during PMS activation. Meanwhile, the terminal-O of PMS was adsorbed on the Co sites of STLC, which promotes the oxidation of PMS to form ^1^O_2_ and SO_4_^•−^. Benefited from the efficient reactive species generation as well as the resistance to various environmental factors, STLC exhibited a great potential to be widely applied in actual environmental remediation. This work not only affords the rational design strategy for Fenton-like catalysts at the atomic scale, but also provides the fundamental insights into the Fenton-like reaction mechanism.

## Results

### Materials synthesis and characterization

**Fig. 1:**
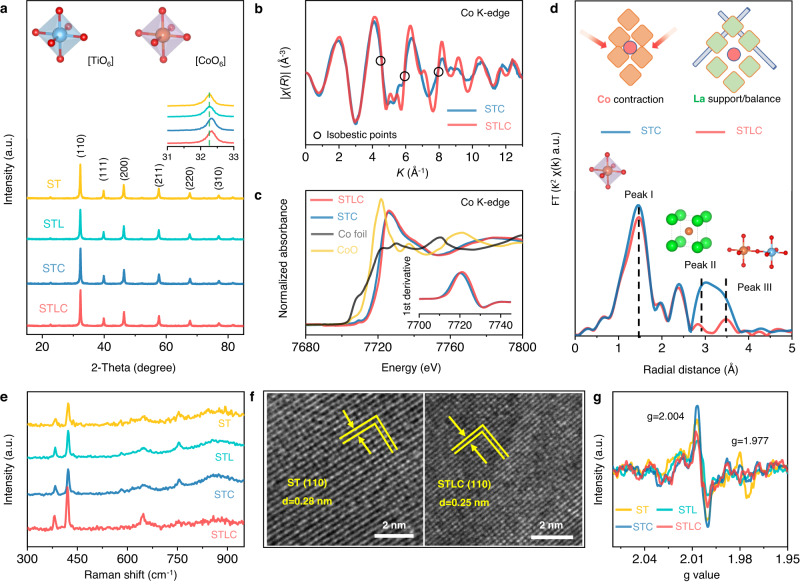
Structural characterizations. **a** XRD patterns of as-prepared catalysts. **b** Co K-edge EXAFS spectra of STC and STLC. **c** Normalized Co K-edge XANES spectra of STC and STLC samples and the standard Co foil and CoO as reference. **d** FT k^3^*χ*(R) Co K-edge EXAFS of STC and STLC. Inset, schematics of the compositions in the STC and STLC. **e** Raman spectrum of as-prepared catalysts. **f** HRTEM images of ST and STLC. **g** EPR spectra of as-prepared catalysts.

The Co/La-SrTiO_3_ perovskites were prepared via a facile liquid-phase reaction method^23^, and four representative samples were chosen: SrTiO_3_ (ST), La-SrTiO_3_ (STL), Co-SrTiO_3_ (STC) and Co/La-SrTiO_3_ (STLC). Among all the samples, the STLC with 0.3% Co and 10% La loading showed the best catalytic activity, and hence it was chosen as the model catalyst in this study. X-ray diffraction (XRD), Fourier transform infrared (FT-IR) spectra, and Raman spectra were initially used to verify the phase structure (Fig. 1a, e and Supplementary Fig. 1). As shown in Fig. 1a, all the diffraction peaks for the synthesized materials are consistent with the standard cubic perovskite structure without the appearance of any impurity phase. Furthermore, the main diffraction peak exhibited a shift to a higher angle with the Co substitution, implying a lattice contraction after the Co doping (Fig. 1a, inset). High-resolution transmission electron microscopy (HRTEM) confirmed the [110] orientation of STLC (Fig. 1f) and the elemental mapping (Supplementary Fig. 2) showed a uniform distribution of all elements^24^. Considering that the redox performance of catalysts in the perovskite structure (ABO_3_) is strongly influenced by B-site transition metals, and the modification of A-sites can significantly change the unit cell structure^25^. Further, the atomic structure changes of A and B sites were characterized.

To further disclose the atomic structure of Co, X-ray photoelectron spectrometry (XPS) and synchrotron radiation-based X-ray absorption spectroscopy (XAS) were performed. The high-resolution Co 2*p* XPS spectra (Supplementary Fig. 3) showed the presence of both Co^2+^ and Co^3+^, and their ratios varied obviously with the modulation of A-site cations (La). As shown in Fig. 1c, the absorption Co K-edge of STLC shifted to higher absorption energy, indicating that the average valence state of Co increased^26^. The edge front region of Co K-edge indicated the electrons transition from 1 *s* to the unoccupied 3*d* orbital, which also clearly assigned to the features of Co_oh_ (octahedral site)^27^. When the local atomic structure of Co was studied by the extended X-ray absorption fine structure (EXAFS), there are some significant isosbestic points, such as 4.5 Å^−1^ and 6.1 Å^−1^ (Fig. 1b), which means a complex property with Co in different components^28^. In addition, the La substitution in STLC also changed the local atomic structure of Co. The attenuation of the peak signal in front of the Co K-edge (Fig. 1c, inset) evidenced a reduction in the overall Co–O octahedron^29^. As shown in Fig. 1d, the two peaks (peak ii and peak iii) of STLC were significantly reduced after La doping, which represented the distance increase of Co-Sr/La and Co–O-Ti, respectively. As observed with Co K-edge EXAFS (Fig. 1d), the first-shell peak of Co–O bond at around 1.5 Å^−1^ and STLC displayed a slight decrease in the Co–O bonds, indicating that there should be more oxygen vacancies (V_o_) in the structure^30^.

To further look into the bonding situation, we extracted Co–O bond lengths and Co coordination numbers from EXAFS curve fitting (Supplementary Fig. 4 and Supplementary Table 1). According to the results, the first shell of the central atom Co showed a coordination number of about 6 (6.0 and 5.8 for Co–O of STC and STLC, respectively), and due to the characteristic of the front peak of the Co K-edge, this also proved that Co atoms have replaced Ti atoms in the B site of the perovskite structure. The results of Raman analysis also showed that all the characteristic vibrational peaks of STLC (383 cm^−1^, 643 cm^−1^, and 752 cm^−1^) were red-shifted, indicating that the bond lengths of different modes were stretched (Fig. 1e). Therefore, the modification of A-site (La) functioned as a stent (Fig. 1d, inset) by making the contracted lattice with Co slightly expand locally, which resulted in the structural balance and change vitality in STLC. Distinct electron distributions between those oxides were further verified using X-band electron paramagnetic resonance (EPR) spectroscopy (Fig. 1g). Electrons accumulated at the Ti center (i.e., Ti^3+^ species, *g* = 1.977) were significantly reduced in STLC compared with bare ST, but the electrons trapping at V_o_ (*g* = 2.003) were increased, indicating more electron transfer from Ti^3+^ species to V_o_ in STLC^31^. The XPS spectrum of Ti also showed a slight shift to higher binding energy, confirming this electron transfer (Supplementary Figs. 5 and 6).

### Catalytic activity and Involved radicals

**Fig. 2:**
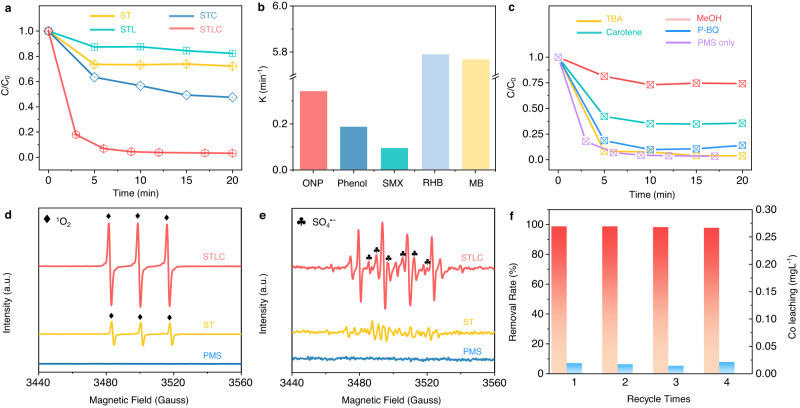
Degradation catalytic performance. **a** ONP removal in different systems. **b** Capability of the STLC system for degrading various pollutants. **c** ONP degradation performance in the presence of radical scavengers. **d**, **e** Spin-trapping ESR spectra of TEMP-^1^O_2_ (**d**) and DMPO-•OH/ SO_4_^•−^ (**e**). **f** Reusability tests of STLC and Co leaching during successive four-time runs.

The Fenton-like catalytic activity of STLC was evaluated by the degradation of ONP and sulfamethoxazole (SMX). The ONP removal rates with different catalysts were compared at an optimal initial pH of 5 in an unbuffered solution (Fig. 2a and Supplementary Fig. 7). Compared with ST, STL, and STC, STLC showed the highest ONP degradation efficiency with a reaction kinetic constant of 0.344 min^−1^. Meanwhile, PMS consumption is much faster in the STLC system (Supplementary Fig. 8a,b). Moreover, a 88.2% TOC removal was achieved in STLC/PMS system, which is five times higher than that of ST/PMS system (17.6%) (Supplementary Figs. 8–10). This result showed that the co-doping of Co and La in STLC enhanced the activation of PMS and removal of organic pollutants. Additionally, as shown in Fig. 2b, STLC also achieved an efficient removal for a wide type of organic micropollutants including SMX, RhB, MB, and phenol.

To explore the major reactive species during the degradation process, chemical quenching experiments were conducted. In the STLC/PMS/ONP system, the addition of methanol (MeOH, a scavenger for •OH and SO_4_^•−^) and β-carotene (^1^O_2_ quencher) can reduce the degradation rate to 21% and 64%, respectively. While the addition of tert-butyl alcohol (TBA, •OH quencher) and p-benzoquinone (p-BQ, O_2_^•−^ quencher) had little effect on the degradation performance (Fig. 2c)^32^. The results of phosphorescence emission, SOSG fluorescence spectrum, and reaction kinetics in D_2_O proved the generation and participation of ^1^O_2_ in the reaction^33^ (Fig. 2d and Supplementary Fig. 11). Furthermore, the EPR analysis results showed that compared with the ST/PMS system, the SO_4_^•−^ and ^1^O_2_ signals were much stronger in the STLC/PMS system (Fig. 2d, e). These results indicated that SO_4_^•−^ and ^1^O_2_ resulting from the STLC-PMS interaction were the main reactive species for ONP removal (Supplementary Fig. 12 and Supplementary Note 1). pH, temperature, and different water matrices (such as Cl^−^, humic acid) have almost no effect on the removal efficiency of ONP, indicating its potential application in the actual complex water environment (Supplementary Figs. 13 and 14). Furthermore, the stability test showed that STLC can maintain high activities during different treatment cycles with a limited Co leaching (Fig. 2f).

### Active site identification and analyzation

**Fig. 3:**
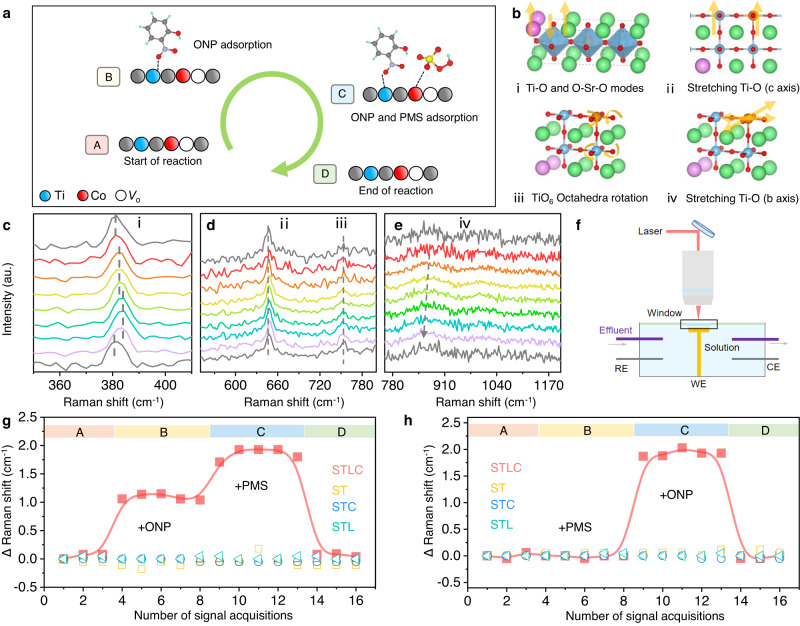
In situ investigation of PMS activation behaviors over different catalysts. **a** The reaction sequence of in situ Raman, first add ONP for a period of time and then add PMS. **b** Vibrational modes of different peaks in the Raman spectrum. **c**–**e** The shift of different peaks in the in situ Raman spectrum. **f** Schematic of the in situ Raman cell set-up. **g**, **h** The movement of the characteristic peak i of the in situ Raman spectra in all samples during the different reaction sequence.

In order to further elucidate the mechanism and the host-guest interaction between STLC and ONP molecule during the reaction, in situ Raman spectroscopy was performed with laser excitation at 632 nm (Fig. 3f and Supplementary Figs. 15–17). The ex situ Raman showed that STLC exhibited Raman peaks at ~383 cm^−1^, ~643 cm^−1^, ~752 cm^−1^, and ~860 cm^−1^, which could be assigned to the O–Sr–O and Co/Ti–O modes, Co/Ti–O stretching frequencies (along “*c* axis”), Co/TiO_6_ octahedral rotation, and Co/Ti–O stretching frequencies (along “*b* axis”), respectively^34^ (Fig. 3b).

Two different in situ reaction sequences were designed to elucidate the unusual catalytic mechanism of STLC (Fig. 3a and Supplementary Fig. 19a). In the first in situ reaction sequence, when ONP was added to the reaction system (stage B), the i peak showed a slight blue shift (Fig. 3c). The ONP concentration decreased during the reaction, indicating that ONP was successfully adsorbed on the STLC surface^35^ (Supplementary Fig. 18). Then a certain concentration of PMS solution was added to the reactor (stage C), peak i showed a further blue shift from 382 cm^−1^ to 384 cm^−1^. Combined with the experimental results of the second in situ reaction sequence, the influence of the dynamic change of STLC structure on the activation process of PMS was explored (Supplementary Fig. 19). As shown in Fig. 3e and Supplementary Fig. 19d, peak iv showed a slight red shift in the two processes. It may be attributed to the surface adsorbed oxygen and more electrons back-donated from Co to the 2*p* antibonding orbital of the adsorbed PMS, resulting in the decrease of the vibration frequency of Co/Ti–O (along “*b* axis”) and the bond length stretch of Co/Ti–O^36^. Peak ii and Peak iii did not undergo any significant changes during the two processes (Fig. 3d and Supplementary Fig. 19c), which indicated that Co/Ti–O stretching frequencies (along “*c* axis”) and Co/TiO_6_ octahedral rotation would not affect the adsorption and activation process of the reactants.

The in situ geometric structure distortion during the Fenton-like catalysis is directly related to the metal centers, which function as a structural response to the adsorption of reaction intermediates^22^. ST, STL and STC were used as the comparative samples for in situ Raman (Supplementary Note 2). Under the same reaction conditions, ST, STL and STC did not significantly change any characteristic peak positions in the two reaction sequences (Fig. 3g, h and Supplementary Figs. 20–31). It was found that doping Co and La alone could not provide any dynamic evolutions on the unit cell, and this extremely stable unit cell resulted in their weak activity in both ONP adsorption and PMS activation. More importantly, Co active sites were prone to self-restructuring on the surface into Co oxyhydroxide during the reaction process, resulting in the loss of active sites^37^. The results of this study showed that all the peaks can restore to the original position after the reaction, and no additional peaks were generated, further proving that STLC maintained good structural stability during the reaction (Fig. 3g, h and Supplementary Fig. 32). The high stability of Co active site could be attributed to the shrinkage of Co–O bond under the practical reaction conditions^38^.

### Correlation of dynamic active site and catalytic activity

**Fig. 4:**
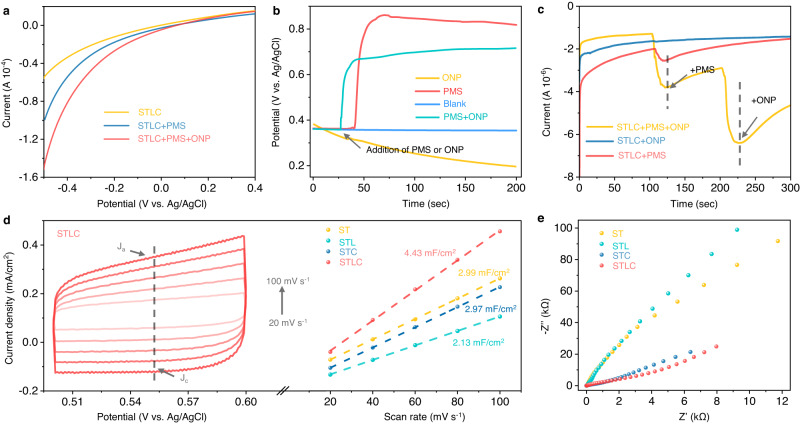
Electrochemically identifying intrinsic activity. **a** LSV curves of STLC under different conditions. **b** Open-circuit potential curves on the STLC electrode in different systems. **c** Amperometric *i–t* curve measurements upon the addition of PMS and ONP using STLC as the working electrode. **d** CV curves of at STLC different scan rates, and ECSA measurements of samples. **e** EIS measurements for different systems.

To elucidate the relationship between the bond length of the unit cell and their catalytic activity, attention was then directed to the effect of dynamic active sites on charge transfer between STLC, PMS, and ONP, electrochemical experiments were carried out. Figure 4a shows the effect of ONP and PMS on the linear sweep voltammetry (LSV) curve of the STLC electrode. The current density of the STLC electrode significantly increased in the presence of PMS, which confirmed that electrons transfer from PMS to the STLC electrode. STLC exhibited a larger anodic current density than ST, STL, and STC, demonstrating there is a greater interface reaction rate in STLC (Supplementary Fig. 33)^39^. The open-circuit potential of STLC sharply rose with the addition of PMS, but the potential gradually decreased with the addition of ONP. While adding PMS and ONP at the same time, the potential of STLC gradually rose but was still lower than the system with only PMS (Fig. 4b). Amperometric *i–t* curves were recorded to monitor charge migration in different systems^40^. When PMS was injected into the STLC system, a negative current appeared immediately, and then the current intensity increased with the addition of ONP (Fig. 4c). It was noticed that in the absence of PMS, the addition of ONP would not cause any change of the current intensity. These results indicated that electrons can be transferred between STLC and PMS during the PMS addition stage A. This process would be significantly promoted after the ONP addition, which also corresponded to the structural change observed by in situ Raman^41^. To further ascertain the electron transfer center on the surface of STLC, the XPS spectrum of the sample after the reaction was measured. As shown in Supplementary Fig. 34, the XPS peaks of Co 2*p* and Ti 2*p* showed a slight shift after the reaction, respectively, while the XPS peaks of other metals (La and Sr) have not changed significantly (Supplementary Fig. 35), suggesting that the adsorption of ONP caused the change of Co/TiO_6_ octahedra in the ABO_3_ structure.

It is necessary to analyze the actual surface area of the catalysts involved in the electrochemical reaction to evaluate their authentic catalytic activity. As shown in Fig. 4d and Supplementary Fig. 36, the corresponding cyclic voltammetry (CV) curves collected after 50 electrochemical activation cycles showed a rectangular CV curve, indicating that the electrochemical behavior originated from the double-layer capacitance (C_dl_)^42^. C_dl_ was approximately calculated based on the charge current density related to the scan rate, and the C_dl_ of STLC (C_dl_ = 4.43 mF cm^−2^) was higher than that of ST, STL, and STC, indicating that it possessed a higher electrochemical active surface area (ECSA)^43^. At the same time, we believe that this is not the reason for the specific surface area (Supplementary Figs. 37 and 38). Moreover, when compared to other samples, the arc radius of the electrochemical impedance spectroscopy (EIS) Nyquist plot of STLC exhibits the smallest arc radius (Fig. 4e), reflecting the lower charge transfer resistance in STLC^44^. These results indicate that the interaction between the reactant molecules (PMS, ONP) and the material is not simply adsorption, but changes the redox ability of STLC. And it further illustrates the strong correlation between the dynamic changes of the structure and the catalytic activity of STLC.

### Theoretical investigations on Fenton-like activity

**Fig. 5:**
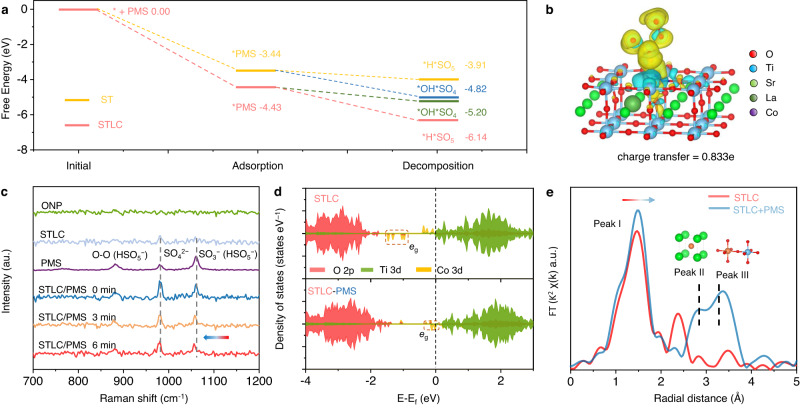
Density functional theory calculation for the PMS activation mechanism. **a** Calculated potential energy diagrams for PMS decomposition into two key reaction intermediates. **b** Adsorption configuration and charge density of PMS on STLC. Yellow and cyan denote electron accumulation and electron depletion, respectively. **c** in situ Raman spectra of ONP, PMS, STLC, and their reaction. **d** Projected density of states of STLC. **e** Co K-edge EXAFS analysis of STLC adsorbed ONP at R space.

With the information of the composition and structure of Co active site, the first-principle density functional theory (DFT) computation was conducted to look into the detailed electronic and geometric structures of the active site during the Fenton-like catalysis (Supplementary Fig. 39). The catalytic reaction pathway in PMS involved several key fundamental steps, including prerequisite PMS adsorption onto the catalysts surface, subsequent dissociation of PMS and generation of reactive species, and concomitant reaction of reactive species with contaminants^45^. Thus, the adsorption sites of PMS/ONP on the STLC surface were first calculated using DFT, indicating that the active sites preferentially adsorbed on O1 sites of PMS, and that PMS and ONP tended to be adsorbed onto Co and Ti sites, respectively (Supplementary Figs. 40 and 41 and Supplementary Table  2). As shown in Fig. 5a, the higher adsorption energy of STLC compared to ST demonstrated its strong binding to PMS, which resulted in its excellent Fenton-like activity. To explore the reactive species generation mechanism, we focused on two important reaction intermediates, which represented different radical generation pathways. The free energy curve of STLC showed that the Bader charge change (0.77 eV) of divided SO_4_* and OH* is smaller than the charge change (1.71 eV) of PMS losing H atoms to generate SO_5_* (Supplementary Fig. 40b). As a result, the PMS-PMS*-SO_5_* path is more thermally favorable, and then SO_5_* rapidly self-decomposed to generate S_2_O_8_^2−^, SO_4_^•−^, and ^1^O_2_^10^. In contrast, PMS favors another pathway in ST, which is PMS-PMS*-SO_4_* and OH*. The excitation wavelength of 532 nm was selected to characterize the change of PMS during the reaction, and the surface mode of the catalyst STLC was relatively weak, which was helpful for testing the chemical bond change of PMS molecules (Fig. 5c). During the reaction, the characteristic vibration of SO_3_^−^ gradually shifts to lower wave numbers, which is due to the decrease in electron density due to the electrons donated by the PMS to the Co sites^46^. This further indicated that PMS was decomposed into SO_5_^•−^ and subsequently ^1^O_2_. This result explained the reason why more ^1^O_2_ and SO_4_^•−^ were generated in the STLC/PMS system.

Furthermore, the internal relationship between the structural changes and the electron transport mechanism during the reaction was investigated. The dynamic change of STLC structure during catalysis is the response of the structure to the adsorption of reaction intermediates, a process highly associated with active metal centers. The predicted densities of states (PDOS) of STLC and ST, as well as their PDOS after adsorption of PMS and ONP, were calculated to explore the active origin of PMS activation. As shown in Supplementary Fig. 42a and Fig. 5d, the PDOS results showed that STLC had a new occupied state dominated by Co 3*d* at the Fermi level compared with ST, resulting in the widening of the total density of states (TDOS) near the Fermi level. This change raised the effective charge concentration on the STLC surface, favoring orbital hybridization between Co 3*d* and O 2*p* of PMS^47^. Furthermore, it was proposed that the *e*_g_ orbital of Co atom may play an important role in enhancing the adsorption strength of reactant molecules (PMS/ONP). After the adsorption of PMS/ONP (Supplementary Fig. 42), the *e*_g_ orbital-occupied state of Co atom moved towards the Fermi level, suggesting *e*_g_ orbital-dominated dynamic catalysis^48^. Meanwhile, as shown in the charge density analysis (Fig. 5b), the charge was redistribution after the chemisorption of PMS/ONP on the STLC surface. The amount of charge transferred from PMS to STLC was increased by about 3-fold compared to ST. In contrast, the amount of charge transferred between Ti sites and ONP was decreased from 0.713e to 0.595e, demonstrating that STLC enhanced the ability of directional electron transfer to PMS during the reaction (Supplementary Table. 3)^49^.

The adsorption-induced bond length shrinkage and stretching directly triggered the refinement of the ligand field around the metal Co atom, which is a prerequisite for achieving efficient electron transfer with the adsorbed oxygen-containing species. DFT calculations showed that the surface Ti–O-Ti lattice spacing from 3.91 to 3.88 Å^−1^, the Co–O bond length changed from 1.92 to 1.97 Å^−1^ when PMS adsorbed onto Co sites of the STLC surface, and the adsorption of ONP molecules also affected geometric deformation of the structure (Supplementary Fig. 43). As shown in the FT-EXAFS spectra in Fig. 5e, STLC showed an increase in peak intensity and a positive shift of the main peak after PMS adsorption, which could be attributed to the formation of new Co–O bonds (Supplementary Fig. 44 and Supplementary Table  1). The intensities of peak ii and peak iii show an increased, indicating the shrinkage of Co-Sr/La and Co–O-Ti in STLC during the reaction, which was consistent with the in situ Raman results. The enhanced white line intensity of STLC/PMS compared with that of STLC suggests the increased density of Co and O 2*p* unoccupied states as a consequence of the strong coupling between STLC and PMS. Furthermore, this shrinkage in the Co–O bond enabled the structure to transition to a lower-energy state, which was thermodynamically favorable for the strain release^19^. This process can further fix Co atoms on the STLC surface, thus avoiding the loss of the active sites during the reaction.

## Discussion

In summary, we have proved that the co doping of La and Co could rationally modify the electronic and surface structure of STLC to promote the dynamic evolutions of the active sites during the Fenton-like catalytic process, thereby achieving a high-speed interfacial charge transfer. Combined experimental and theoretical studies revealed that the reversible vibrational contraction and stretching of O–Sr–O and Ti–O bonds in different orientations in STLC were induced by adsorbed PMS and ONP molecules. Subsequently, this optimized structural distortion was beneficial for modulating the electronic structure and enhanced the metal-oxygen bond strength of the metal active sites in STLC, thereby facilitated electron transfer from PMS to Co. Meanwhile, the reactive species generation mechanism during the reaction was is explained as the Co sites in STLC adsorbed the terminal-O of PMS and promoted the oxidation of PMS to form SO_5_^•−^. STLC thus exhibited outstanding intrinsic activity and stability toward the Fenton-like activation. Its high performance has also been demonstrated in various types of organics removal as well as different water matrix. On the basis of this work, we explored new strategies for adjusting the chemical activity of active sites by designing dynamic structures to develop advanced materials for heterogeneous catalysis.

## Methods

### Synthesis of catalysts

Typically, 2.48 mmol of C_16_H_36_O_4_Ti, 2.23 mmol Sr(CH_3_COO)_2_•0.5H_2_O and 0.25 mmol La(NO_3_)_3_•6H_2_O were slowly added into 30 mL of ethanol solution and agitated for 1 h, to which 0.25 g NaOH was added and stirred for another 1 h. 0.07 mmol Co(NO_3_)_2_•6H_2_O precursor was introduced into the above solution. The mixture was then transferred into a Teflon autoclave (100 mL) and kept at 220 °C for 72 h. The obtained products were collected by centrifugation and washed three times by deionized water and ethanol, and dried in a vacuum oven at 60 °C for 12 h. Finally, the obtained products were milled and sintered at 800 °C for 2 h in a muffle furnace to get the nanocrystalline Co/La-SrTiO_3_ powder.

### Characterizations

The powder X-ray diffraction (XRD) was recorded on a Rigaku D/Max 2200PC X-ray diffractometer with Cu Kα radiation (*λ* = 0.15418 nm). X-ray photoelectron spectroscopy (XPS) was performed on Thermal ESCALAB 250 electron spectrometers. The elemental content of the samples was determined through the ICP-MS (X7 Series, Thermo Electron Corporation). Fourier transform infrared (FT-IR) spectra is obtained on a Nicolet 6700 spectrophotometer (Thermofisher). Electron paramagnetic resonance (EPR) spectrometer (A300-10/12, Bruker) was used to observe the generated reactive oxygen species. Total organic carbon (TOC) was measured via an Analytikjena multi N/C analyzer. The Brunauer–Emmett–Teller (BET) specific surface areas and pore-size distributions of the samples were analyzed using a Micromeritics ASAP 2460 system at liquid nitrogen temperature. The in situ Raman was collected using a confocal Raman microscope (ThermoScientificDXR2) with an excitation wavelength of 632 nm and 5.0 mW Laser power. Each Raman spectrum was acquired over an exposure time of 0.05 s and is the number of scans is 50. Electrochemical characterizations were performed with a CHI760E bipotentiostat with a standard three-electrode configuration.

### Analysis methods

The performance of the catalysts for PMS activation and pollutant degradation were evaluated by using o-nitrophenol (ONP) and (sulfamethoxazole) SMX as a model recalcitrant pollutant. All experiments are conducted on the XPA-7 chemical reactor (Nanjing Xujiang Electromechanical Plant) at 25 °C. In all, 1.0 g L^−1^ catalyst and 0.4 g L^−1^ PMS are added into 60 mL of pollutant solution (20 mg L^−1^). The free radicals (such as •OH, SO_4_^•−^, and O_2_^•−^) and single oxygen (^1^O_2_) were recorded on electron paramagnetic resonance (EPR) spectrometer (A300-10/12, Bruker) with 60.0 mM of 5,5-dimethyl-pyrroline N-oxide (DMPO) and 2,2,6,6-tetramethyl-4-piperidinol (TEMP) as the spin-trapper agent, respectively. The degradation intermediates of SMX were identified by ultraperformance liquid chromatography−high-resolution mass spectrometry (UPLC − HRMS, Orbitrap Fusion, Thermo, USA). The eluent was composed of 0.1% methanoic acid and acetonitrile, and the flow rate was 0.2 mL/min. All the experiments were carried out in triplicate.

### X-ray absorption spectroscopy measurements

The Co K-edge X-ray absorption fine structure spectra of STLC and STC and its references were obtained at 1W1B station in Beijing Synchrotron Radiation Facility (BSRF) under ambient conditions using a transmission mode. The storage rings of BSRF were operated at 2.5 GeV with a maximum current of 250 mA. Using Si (111) double-crystal monochromator, the data collection was carried out in transmission mode using an ionization chamber. All spectra were collected at ambient conditions.

### Calculation details

We performed the first-principles calculations in the frame of density functional theory (DFT) with the Vienna ab initio simulation package (VASP). The exchange-correlation energy is described by the Perdew–Burke–Ernzerhof (PBE) form of generalized-gradient approximation (GGA) exchange-correlation energy functional. The structure optimization of SrTiO_3_ was carried out by allowing all atomic positions to vary and relaxing lattice parameters until the energy difference of successive atom configurations was less than 10^−4^ eV. The PMS and ONP molecules were put in 10 Å × 10 Å × 10 Å lattice. The force on each atom in the relaxed structures was less than 0.015 eV/Å. The cutoff energy for the plane-wave basis set was set to 400 eV. The k-point spacing was set to be smaller than 0.03 Å^−1^ over the Brillouin zone (BZ).

## Supplementary information

Supplementary Information

## Data Availability

The data that support the findings of this study are available from the corresponding author upon reasonable request.
