# Supplementary material for: Dynamic active-site induced by host-guest interactions boost the Fenton-like reaction for organic wastewater treatment
Source: Nat Commun. 2023 Jun 15;14:3538. doi: 10.1038/s41467-023-39228-4 (PMC10272134; doi:10.1038/s41467-023-39228-4)
Supplement: Supplementary file 1 — Supplementary Information [file 41467_2023_39228_MOESM1_ESM.pdf]

Dynamic active-site induced by host-guest interactions boost the Fenton-like reaction for organic wastewater treatment

Dongpeng Zhang[1,#], Yanxiao Li[1,#], Pengfei Wang[2], Jinyong Qu[1], Yi Li[3,4*] and Sihui Zhan[1*]

[1]Key Laboratory of Pollution Processes and Environmental Criteria (Ministry of Education), College of Environmental Science and Engineering, Nankai University, Tianjin, China.

[2]Tianjin Key Lab Clean Energy & Pollutant Control, School of Energy and Environmental Engineering, Hebei University of Technology, Tianjin 300130, China

[3]Tianjin Key Laboratory of Molecular Optoelectronic Sciences, Department of Chemistry, School of Science, Tianjin University & Collaborative Innovation Center of Chemical Science and Engineering (Tianjin), Tianjin 300072, China

[4]Joint School of National University of Singapore and Tianjin University, Fuzhou International Campus, Tianjin University, Binhai New City, Fuzhou 350207, China

[#]These authors contributed equally: Dongpeng Zhang, Yanxiao Li.

Correspondence to: Sihui Zhan (sihuizhan@nankai.edu.cn); Yi Li (liyi@tju.edu.cn)

This PDF file includes:

Supplementary Methods

Supplementary Figures 1 to 44

Supplementary Tables 1 to 3

Supplementary Notes 1 to2

Supplementary Methods

Methods

Synthesis of $SrTiO_3$: In the typical procedure, 2.48 mmol of $C_{16}H_{36}O_4Ti$ and 2.23 mmol $Sr(CH_3COO)_2$•$0.5H_2O$ were slowly added into 30 mL of ethanol solution and agitated for 1 h, to which 0.25 g NaOH was added and stirred for another 1 h. The mixture was transferred into a Teflon autoclave (100 mL) and kept at 220 °C for 72 h. The obtained products were collected by centrifugation and washed three times by deionized water and ethanol, and dried in a vacuum oven at 60 °C for 12 h. Finally, the obtained products were milled and sintered at 800 °C for 2 h in a muffle furnace to get the nanocrystalline $SrTiO_3$ powder.

Synthesis of Co-$SrTiO_3$: Typically, 2.48 mmol of $C_{16}H_{36}O_4Ti$ and 2.23 mmol $Sr(CH_3COO)_2$•$0.5H_2O$ were slowly added into 30 mL of ethanol solution and agitated for 1 h, to which 0.25 g NaOH was added and stirred for another 1 h. 0.07 mmol $Co(NO_3)_2$•$6H_2O$ precursors was introduced into the above solution. The mixture was transferred into a Teflon autoclave (100 mL) and kept at 220 °C for 72 h. The obtained products were collected by centrifugation and washed three times by deionized water and ethanol, and dried in a vacuum oven at 60 °C for 12 h. Finally, the obtained products were milled and sintered at 800 °C for 2 h in a muffle furnace to get the nanocrystalline Co-$SrTiO_3$ powder.

Synthesis of La-$SrTiO_3$: 2.48 mmol of $C_{16}H_{36}O_4Ti$, 2.23 mmol $Sr(CH_3COO)_2$•$0.5H_2O$ and 0.25 mmol $La(NO_3)_3$•$6H_2O$ were slowly added into 30 mL of ethanol solution and agitated for 1 h, to which 0.25 g NaOH was added and stirred for another 1 h. The mixture was transferred into a Teflon autoclave (100 mL) and kept at 220 °C for 72 h. The obtained products were collected by centrifugation and washed three times by deionized water and ethanol, and dried in a vacuum oven at 60 °C for 12 h. Finally, the obtained products were milled and sintered at 800 °C for 2 h in a muffle furnace to get the nanocrystalline La-$SrTiO_3$ powder.

Adsorption energy

To calculate the adsorption energies of PMS and ONP molecules on $SrTiO_3$ (001) surface with oxygen-vacancy and La, Co co-doped $SrTiO_3$ (001) surface with oxygen-vacancy, we first construct the corresponding surfaces with 6 layers. In order to obtain the most stable structures, their structure optimizations were performed by fixing bottom 4 layers and setting the thickness of 15 Å vacuum. Then, the adsorption energies were calculated by the following equation:

$$\Delta E_{ad} = E_{system} - E_{surface} - E_{PMS\ or\ ONP} \quad (1)$$

Where, E_{system}, $E_{surface}$ and $E_{PMS\ or\ ONP}$ represent the total energies of adsorption system, surface and PMS or ONP molecule, respectively. The calculated results are shown in Table S2.

Charge difference density of interface system

To intuitively observe the adsorption binding, the charge density difference (CDD) of PMS and ONP molecules on $SrTiO_3$ (001) surface with oxygen-vacancy and La, Co co-doped $SrTiO_3$ (001) surface with oxygen-vacancy were calculated according to

$$\Delta\rho = \rho_{system} - \rho_{surface} - \rho_{molecule} \quad (2)$$

where ρ_{system} is the charge density of the adsorption system and $\rho_{surface}$ and $\rho_{molecule}$ are the charge densities of surface and PMS or ONP molecule, respectively.

Electrochemical Measurement

The CV and ECSA properties of catalyst were tested in a three-electrode cell using an electrochemical analyzer and rotating disk electrode (CHI760E). The catalyst loaded glassy carbon-rotating disk electrode (GC-RDE, diameter of 5 mm) was used as a working electrode. The catalyst ink was prepared by sonication of 10 mg catalyst powder, 400 μL ethanol and 50 μL Nafion solution (10 wt%). Then, the approximate amount of catalyst ink was loaded onto GC-RDE. The catalyst layer was dried in ambient air before use. The Pt foil electrode and Ag/AgCl were used as a counter and reference electrode,

respectively. The amperometric i-t curve and open-circuit potential properties of catalyst were tested in a three-electrode cell using an electrochemical analyzer and rotating disk electrode (CHI760E). The Pt foil electrode and Ag/AgCl were used as a counter and reference electrode, respectively. The linear sweep voltammetry (LSV) experiments were measured in a 0.1 M Na_2SO_4 solution between 0 and -1.0 V vs RHE at a scan rate of 5 mV s^{-1}.

Singlet Oxygen ($^1O_2$) Detection.

SOSG was selected to detect the generated $^1O_2$. During the reaction, the mixture solution of 2-mL aliquots was collected at a given interval and then 2 μL SOSG (5 μM) was added. The $^1O_2$ generation can be estimated by PL intensity increase of the above mixture, where 504 and 525 nm were chosen as the excitation and emission wavelengths, respectively. The $^1O_2$ 1270 phosphorescence emission was measured on FLS1000 time-resolved photoluminescence spectrometer. To achieve a sufficient signal-to-noise ratio, the singlet oxygen luminescence counts were summed over 28.5 s.

Supplementary Figures

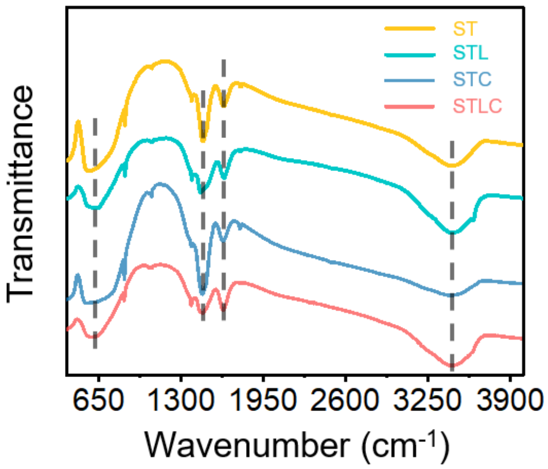

Supplementary Fig. 1. The FTIR spectra of ST, STL, STC, STLC.

The low-frequency absorption bands around 600 cm^{-1} indicated in Supplementary Fig. 1 corresponded to the symmetric and asymmetric stretching modes of Ti-O bond within the TiO_6 octahedron cluster. The band located around 3500 cm^{-1} corresponded to OH hydration groups. The asymmetry resulting from the increase in intensity of this band in the low frequency side (3500-3250 cm^{-1}) indicated the formation of hydrogen bonds. The peaks of all samples were consistent, indicating that the structure of the doped samples did not change significantly from that of the substrate.

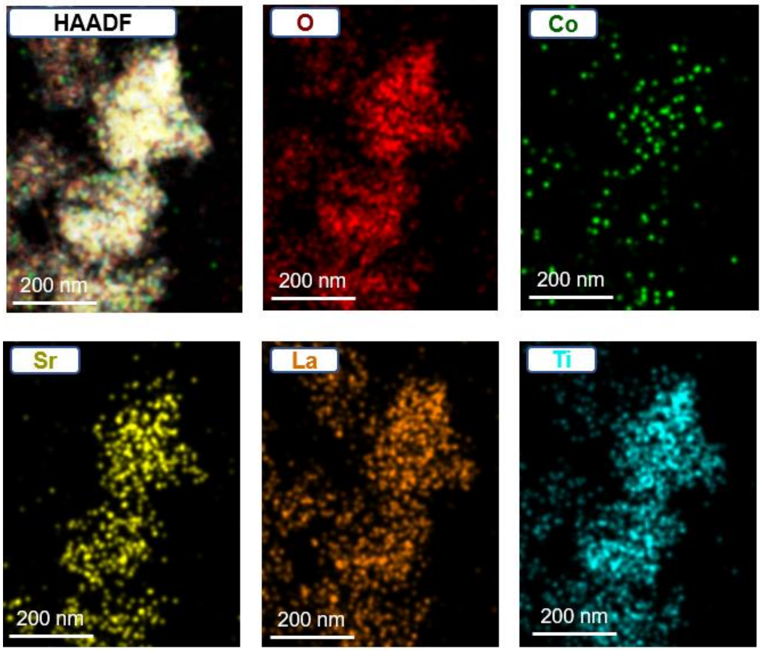

Supplementary Fig. 2. EDS elemental mapping images of STLC.

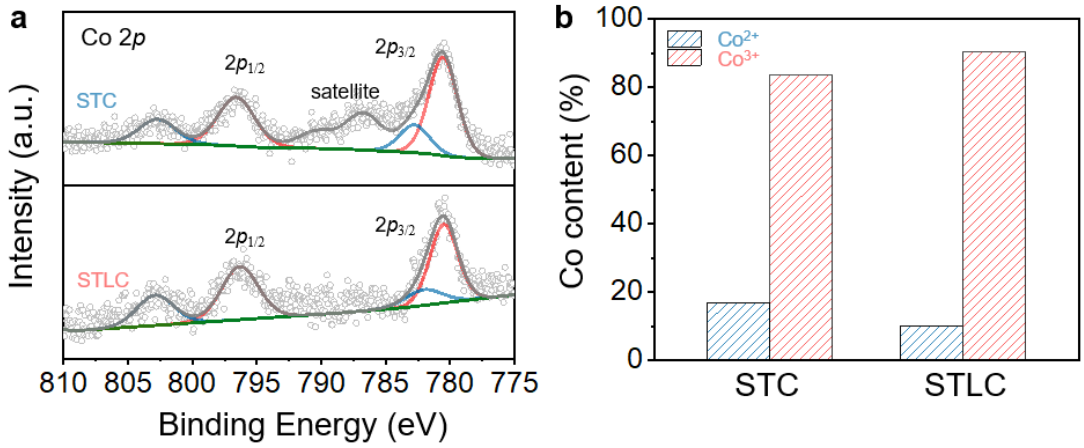

Supplementary Fig. 3. a The Co 2*p* spectra of STC, STLC, **b** the Contents of Co^{2+} and Co^{3+} in STC and STLC.

The content of Co^{2+} and Co^{3+} in STLC is 9.8% and 90.2%, respectively. The content of Co^{2+} and Co^{3+} in STC is 16.68% and 83.32%, respectively. It can be seen that the doping of La increased the ratio of Co^{3+}.

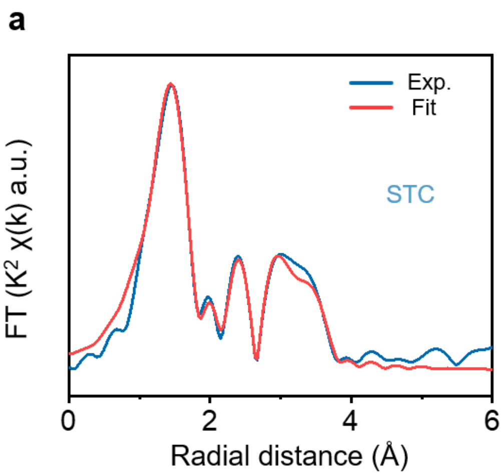

Supplementary Fig. 4. EXFAS R space fitting curve (red line) and the experimental data of STC **a** and STLC **b**.

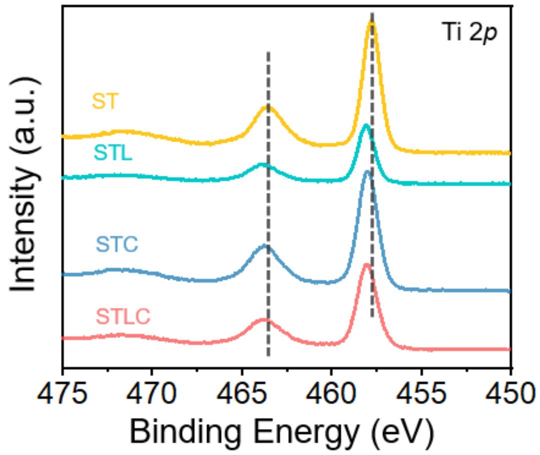

Supplementary Fig. 5. The Ti 2*p* spectra of samples.

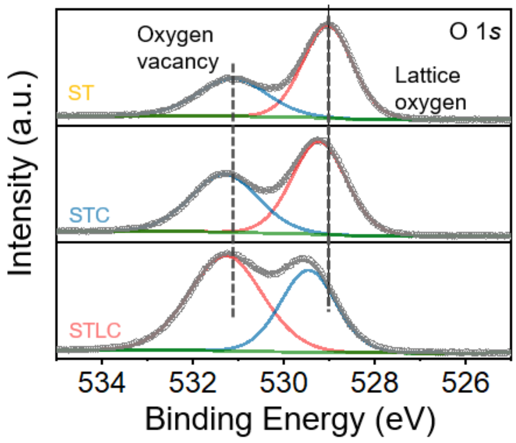

Supplementary Fig. 6. The O 1*s* spectra of ST, STC, STLC.

The high-resolution O 1*s* spectra can be indexed into two main distinctive binding energy peaks centered at 529.0 eV and 531.2 eV, which could be attributed to the lattice oxygen and vacancy oxygen. The proportions of oxygen vacancies in ST, STC, STLC are 35.97, 43.72 and 60.36, respectively. It can be seen that the incorporation of Co and La greatly increased the content of vacancy oxygen.

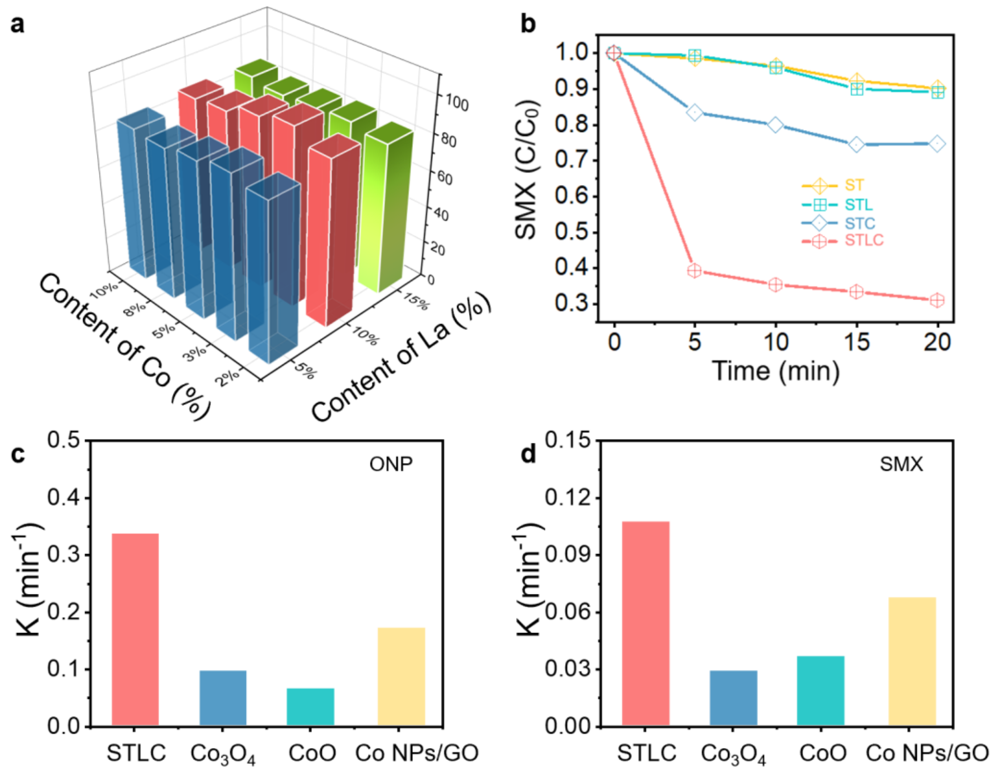

Supplementary Fig. 7. a The catalytic performance of catalysts with different Co and La contents in 5 minutes. **b** Degradation of SMX by different materials. Reaction conditions: initial pollutant 20 mg/L, initial PMS 0.4 g/L, catalyst 1.0 g/L. **c** The ability of STLC and other Co-based catalysts to degrade ONP. **d** The ability of STLC and other Co-based catalysts to degrade SMX.

As shown in Supplementary Fig. 7a, we determined that the optimal doping amounts of Co and La in the material were 3% and 10%, respectively. Furthermore, as shown in Fig. 2a, the presence of Co, La doping resulted in the significantly higher catalytic activity of STLC than STC, suggesting that the simultaneous modification of A and B sites enhanced the catalytic activity and might bring new changes to the active sites. La doping was a common and effective method for modifying the A site of perovskite. It had been shown that La doping contributed to the generation of dynamic active sites of catalysts in some reactions. As shown in Supplementary Fig. 7c and Supplementary Fig. 7d, we compared the ability of STLC to degrade ONP and SMX with other common Co-based catalysts, and the results showed that STLC had good catalytic activity.

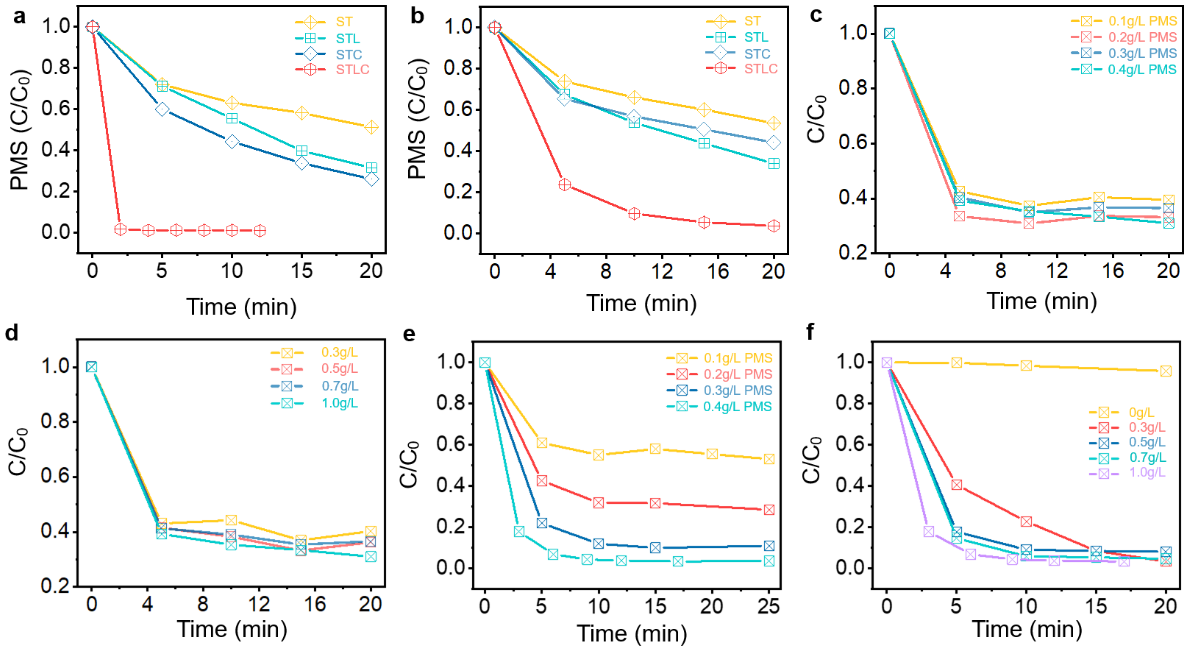

Supplementary Fig. 8. a PMS decomposition curves in different material system with ONP, **b** PMS decomposition curves in different material system without ONP, **c** dosage of PMS (initial pollutant 20 mg/L, catalyst 1.0 g/L), **d** dosage of STLC (initial pollutant 20 mg/L, initial PMS 0.4 g/L), **e** dosage of PMS (ONP 20 mg L^{-1}, STLC 1.0 g L^{-1}), **f** dosage of STLC (ONP 20 mg L^{-1}, PMS 0.4 g L^{-1}).

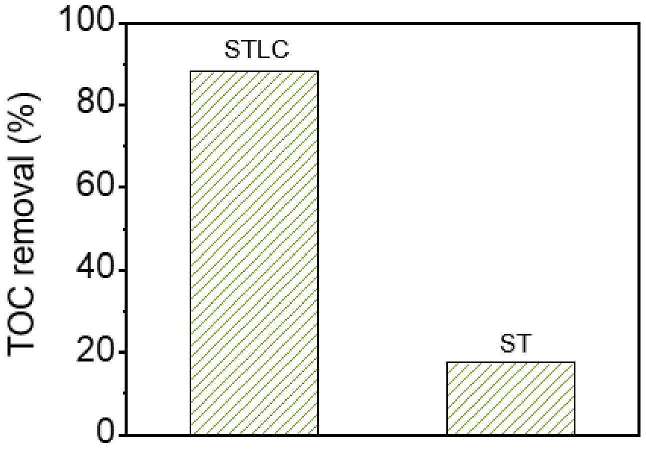

Supplementary Fig. 9. TOC removal curves during ONP degradation in 1 h.

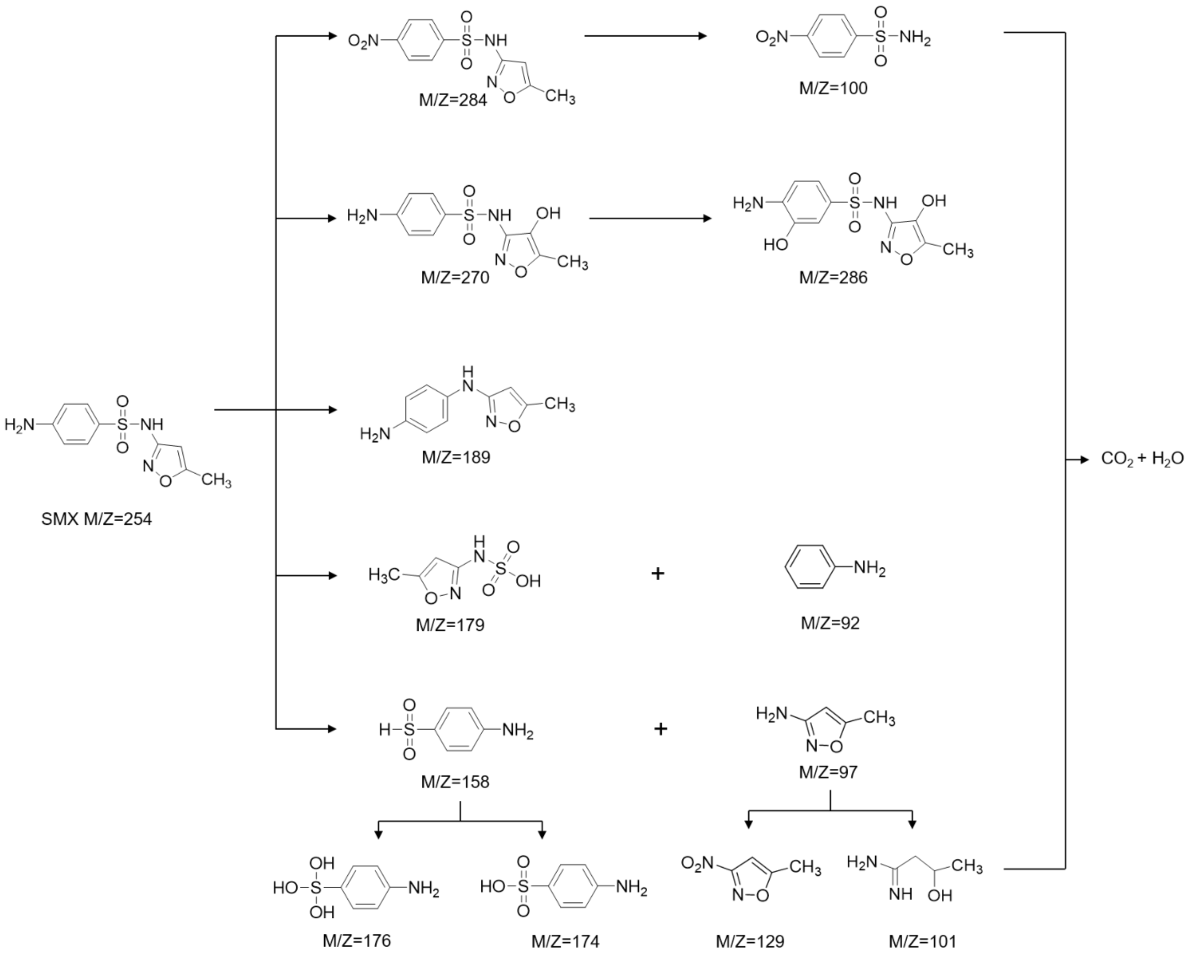

Supplementary Fig. 10. Proposed pathway of SMX degradation under STLC/PMS/SMX system.

This degradation pathway is based on the analysis of the major peaks in the LC-MS chromatograms. Other non-detected reaction intermediates might also exist.

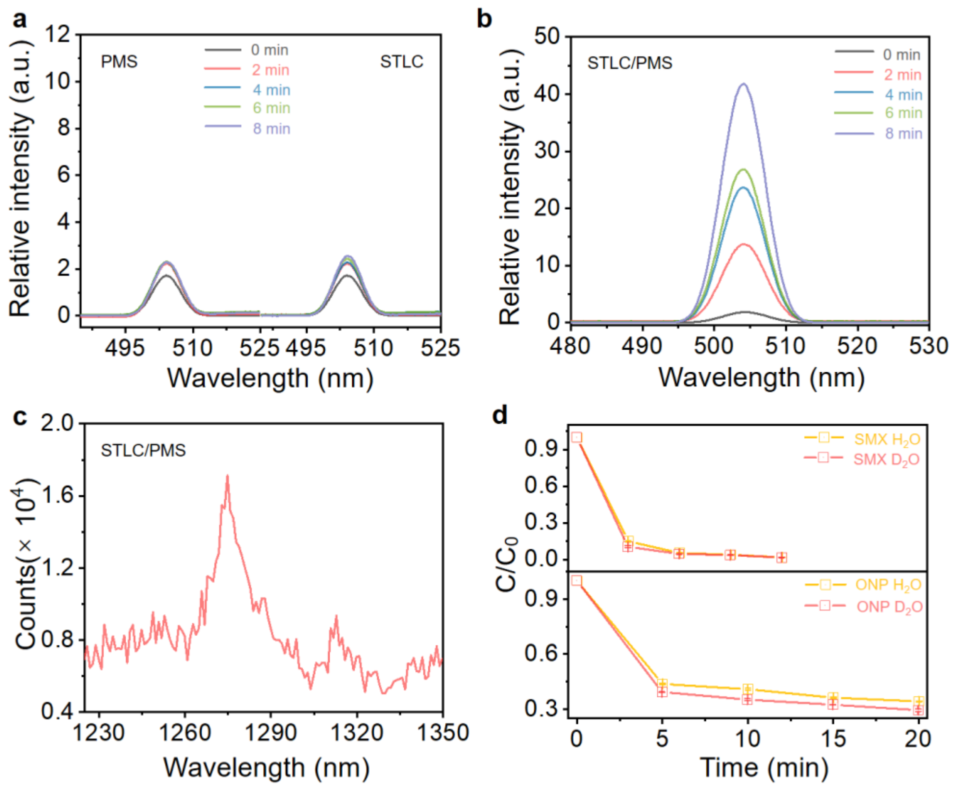

Supplementary Fig. 11. a Time-dependent fluorescence intensity in the reaction of SOSG with $^1O_2$ generated in PMS and STLC systems, **b** Time-dependent fluorescence intensity in the reaction of SOSG with $^1O_2$ generated in PMS/STLC system, **c** The 1270 nm phosphorescence emission of $^1O_2$ in H_2O, **d** The comparison of degradation efficiency of ONP and SMX in H_2O and D_2O.

As shown in Supplementary Fig. 11a, in the case of only PMS and STLC, a weak fluorescent signal was observed in the reaction system. The signal intensity remained unchanged with the prolongation of reaction time. In the PMS/STLC system, the fluorescence signal was obvious and gradually increased with the reaction, indicating the stable formation of $^1O_2$ (Supplementary Fig. 11b). As shown in Supplementary Fig. 11c there was a center-centered sharp peak around at ~1270 nm, corresponding to the physical quenching of $^1O_2$, verifying the generation of $^1O_2$ in the reaction system. We hope to further determine the participation of $^1O_2$ in the degradation by comparing the reaction efficiency in D_2O and H_2O. But because the activation of PMS in D_2O environment is more difficult than in H_2O environment, resulting in less activation of PMS (Supplementary Fig. 11d). This result also suggests that the effect of D_2O on the lifetime of $^1O_2$ may not apply to all systems.

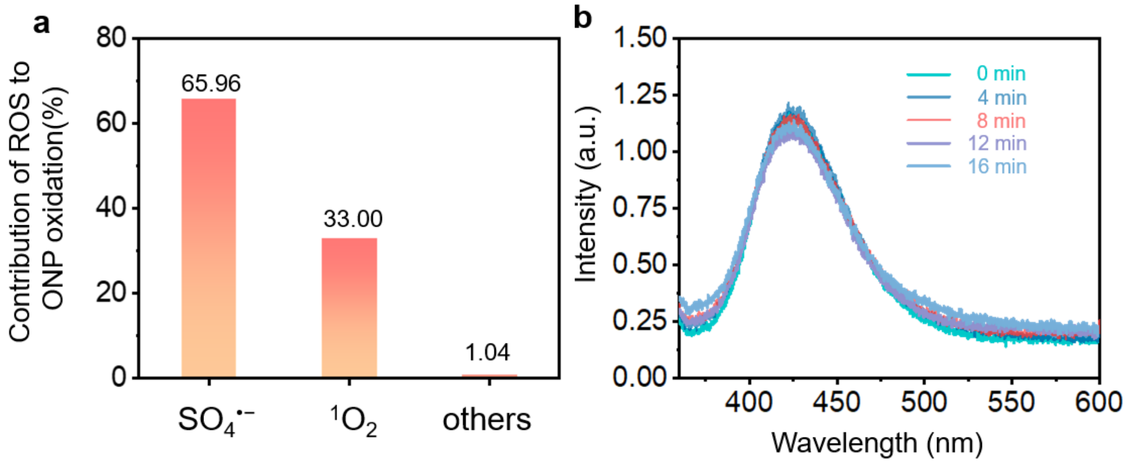

Supplementary Fig. 12. a The contribution of free radicals. **b** PL intensity of TA-OH in STLC activated PMS system.

In order to eliminate the contribution of •OH, we conducted the free radical contribution analysis and the PL fluorescence test of •OH. The contribution from •OH and other radical reactions was very low in the reaction system. Further, active species of •OH was not detected during the reaction. Specifically, the absorption intensity the photoluminescence (PL) intensity of 2-hydroxy terephthalic (TA-OH) at 425 nm (the probe for •OH) remained constant throughout the reaction. These evidences rule out the contribution of •OH during the degradation process.

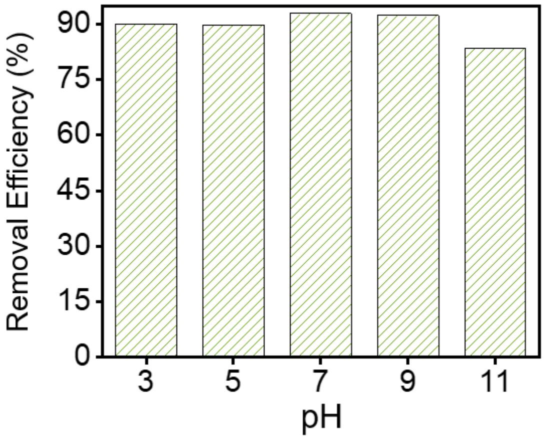

Supplementary Fig. 13. Under different pH, the removal rate of ONP in STLC/PMS/ONP system.

To optimize the removal rate of ONP by STLC, the effects of key reaction parameters such as catalyst dosage, PMS dosage, pH and temperature on ONP degradation rate were studied (SMX optimization research is shown in Supplementary Fig. 10 and Supplementary Fig. 11). When the addition amount of catalyst and PMS is fixed at 1.0 g L^{-1} and 0.4 g L^{-1}, the highest degradation efficiency can be achieved. Increasing the dosage of PMS and catalyst hardly improves the degradation efficiency, and affects the economy in the actual application process (Supplementary Fig. 12).

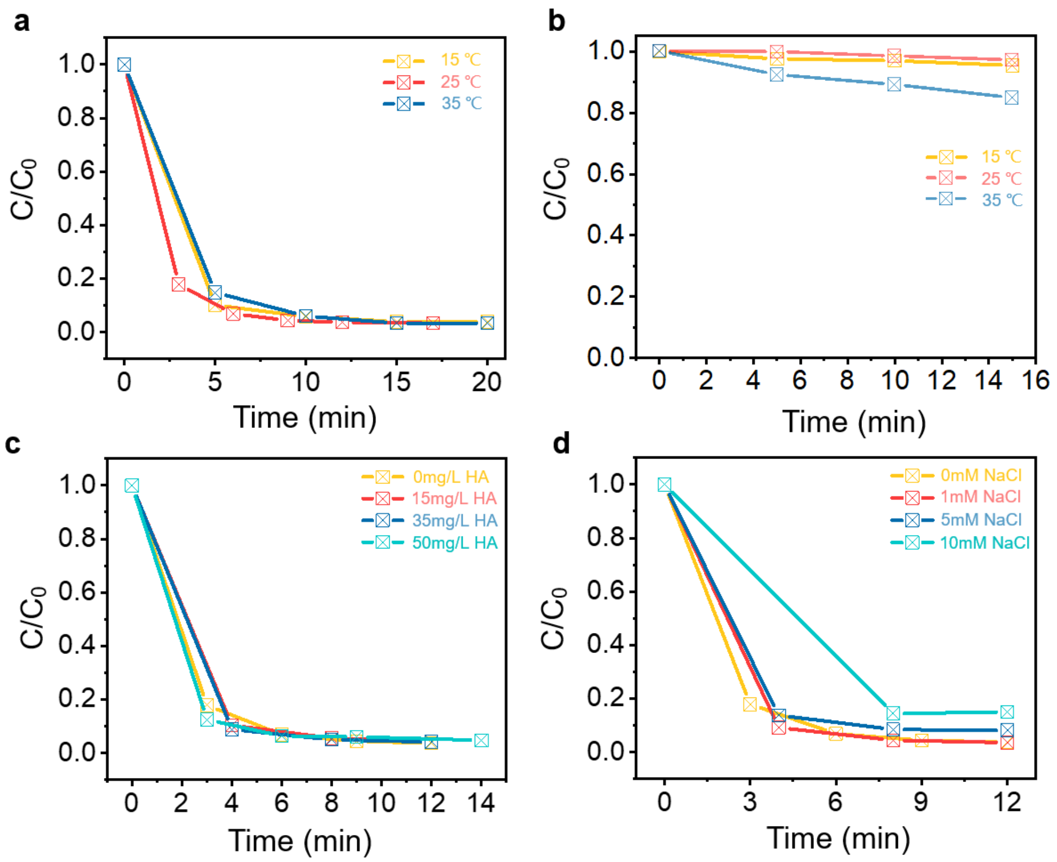

Supplementary Fig. 14. Different influencing factors of the STLC/PMS/ONP system: **a** Different temperatures, **b** without STLC, the effect of different temperatures, **c** different concentrations of HA, **d** different concentrations of NaCl.

Temperature, HA and Cl^{-} have no obvious effect on the degradation rate.

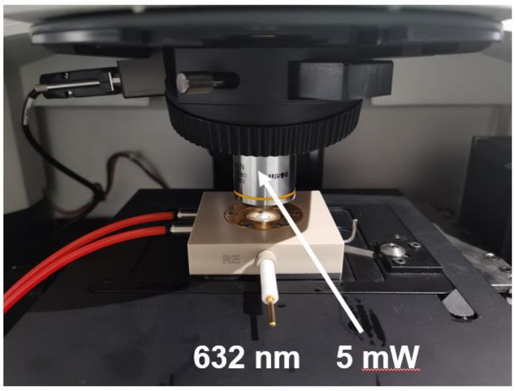

Supplementary Fig. 15. The image of in situ Raman spectroscopy employed in the study.

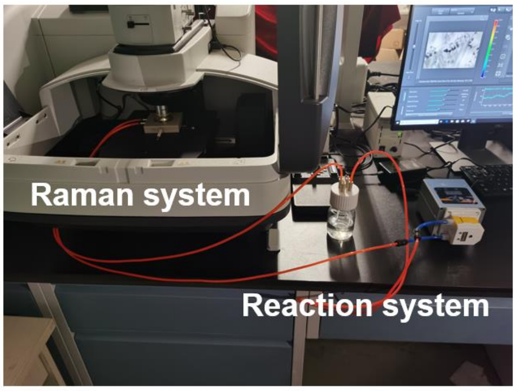

Supplementary Fig. 16. The schematic diagram of in situ Raman and reaction system.

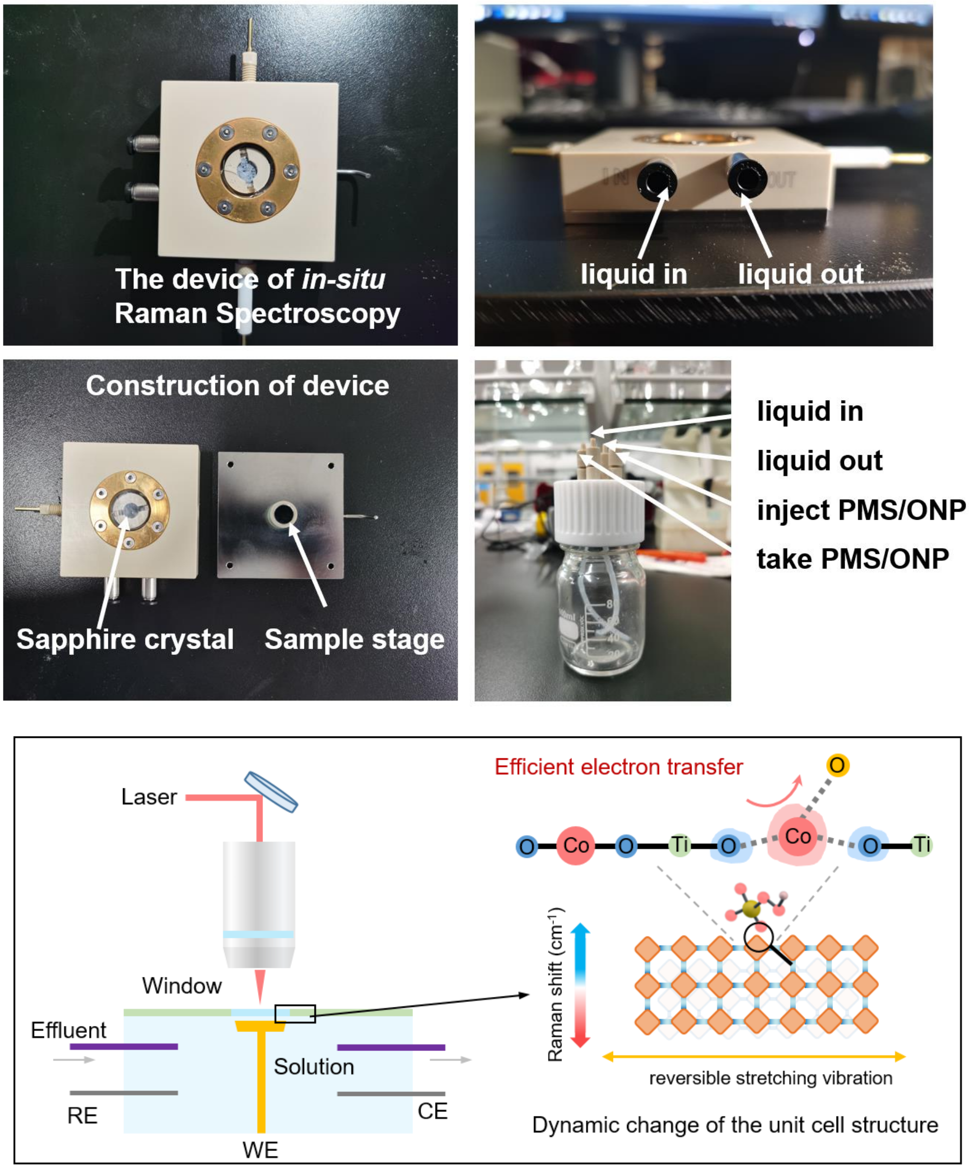

Supplementary Fig. 17. The schematic diagram of in situ reaction tank and circulation device structure.

Supplementary Fig. 15, 16 and 17 are in situ Raman reaction diagrams. Materials need to be tested after air drying at the sample stage. PMS/ONP was added in the measurement process to explore the change of Raman spectrum in the reaction process. And the amount of catalyst was appropriately reduced to obtain a longer reaction time in order to study the reaction process in detail. CE, counter electrode; RE, reference electrode; WE, working electrode.

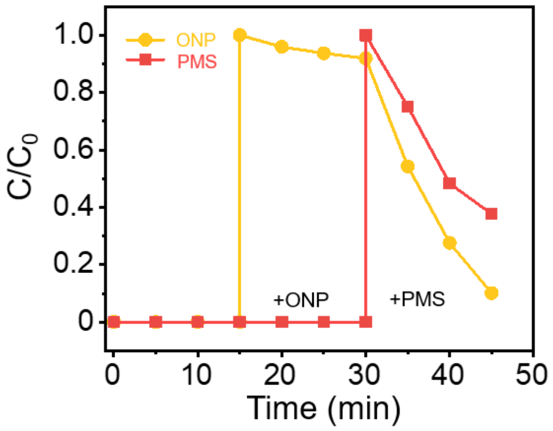

Supplementary Fig. 18. The concentrations of PMS and ONP change during the reaction, ONP is added first and PMS is added later.

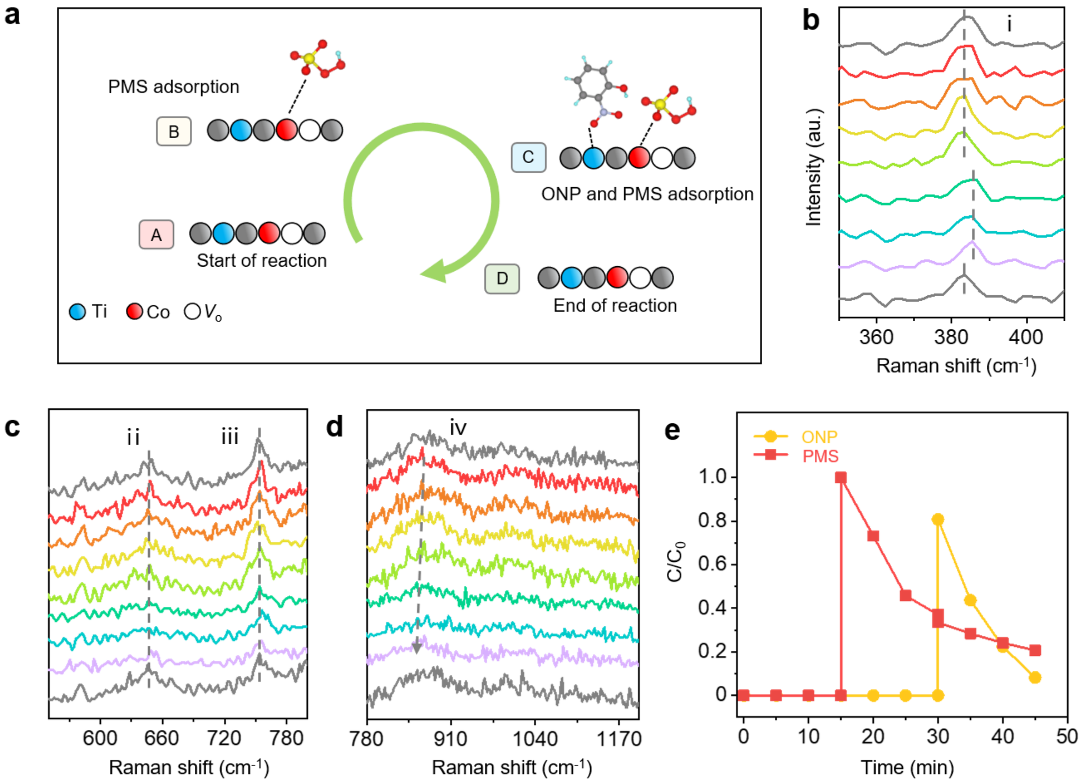

Supplementary Fig. 19. a,**b**,**c**,**d** The different reaction stages of STLC were originally Raman spectra, **e** the concentration of PMS and ONP changes during the reaction, PMS is added first and ONP is added later.

As shown in Supplementary Fig. 19b, peak i did not move in both the initial stage (stage A) and the PMS addition stage (stage B), even though STLC had already started to adsorb and activate PMS at this stage (Supplementary Fig. 19e). Further adding a certain concentration of ONP solution, the violent catalytic reaction at the interface caused peak i to move to the same position as the C stage in the first reaction sequence, indicating that the adsorption of ONP on STLC induced the bond length contraction of O-Sr-O and Co/Ti-O modes. And this tendency is obviously enhanced when electrons are transferred from PMS to STLC, which may benefit PMS activation and reaction acceleration.

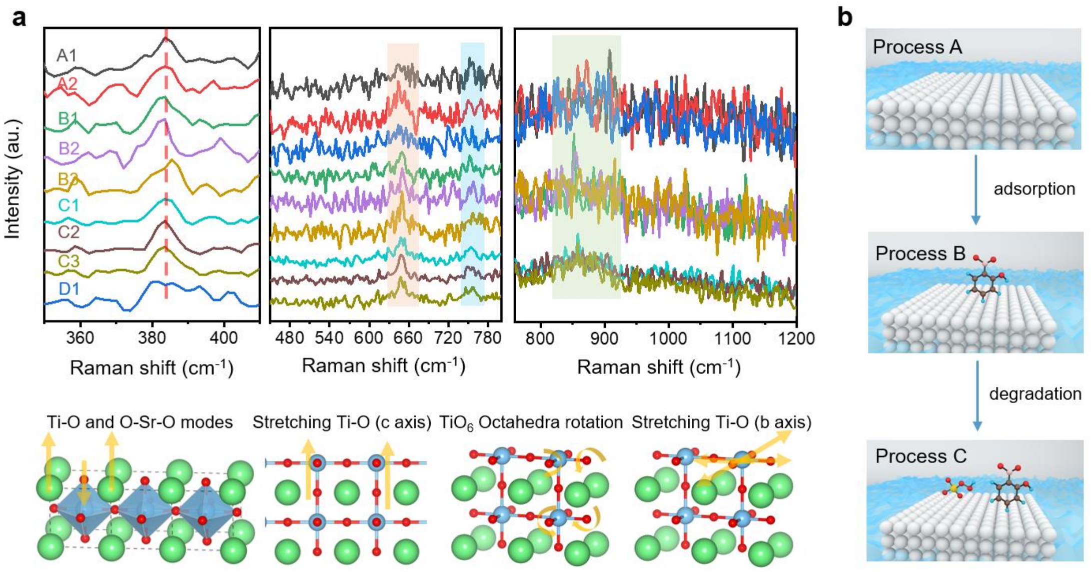

Supplementary Fig. 20. a In situ Raman spectra of ST at different reaction stages, corresponding to **b**. **b** The reaction sequence of in situ Raman, first add ONP for a period of time and then add PMS.

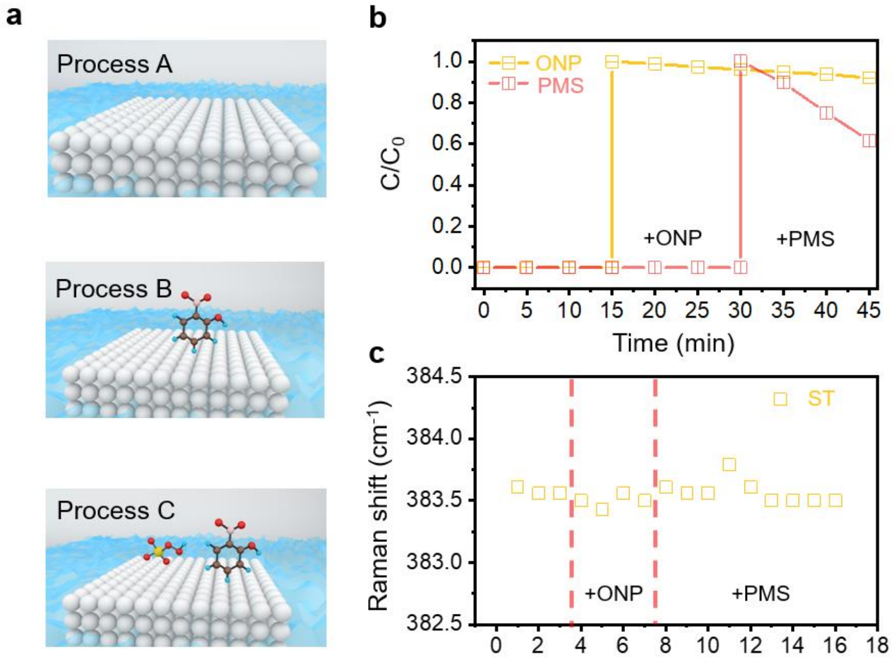

Supplementary Fig. 21. a The reaction sequence of ST of in situ Raman, first add ONP for a period of time and then add PMS. **b** The change of reactant concentration. **c** The change of the Raman shift of peak i.

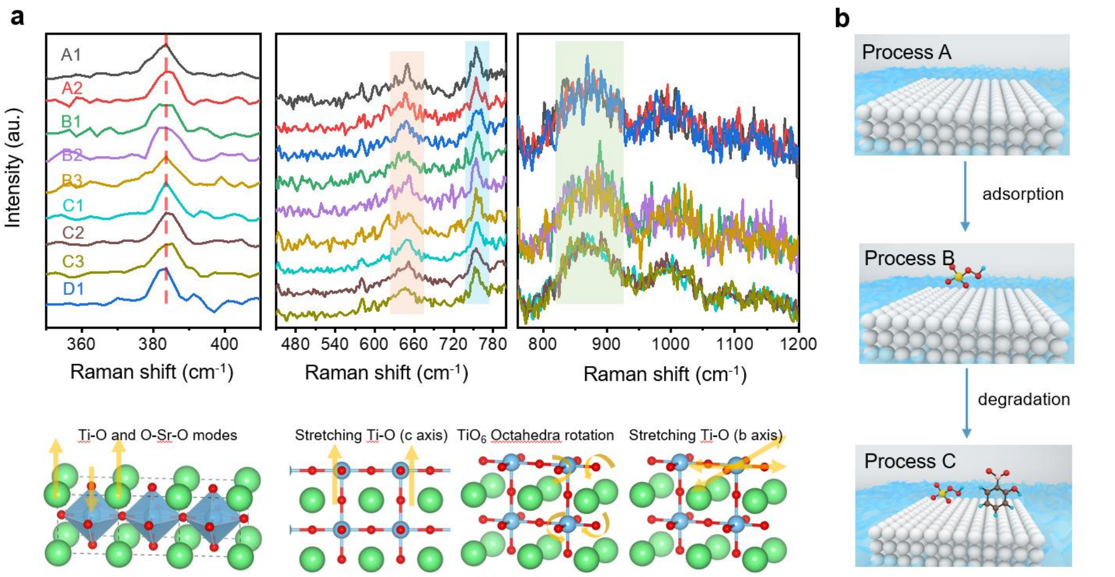

Supplementary Fig. 22. a In situ Raman spectra of ST at different reaction stages, corresponding to **b**. **b** The reaction sequence of in situ Raman, first add PMS for a period of time and then add ONP.

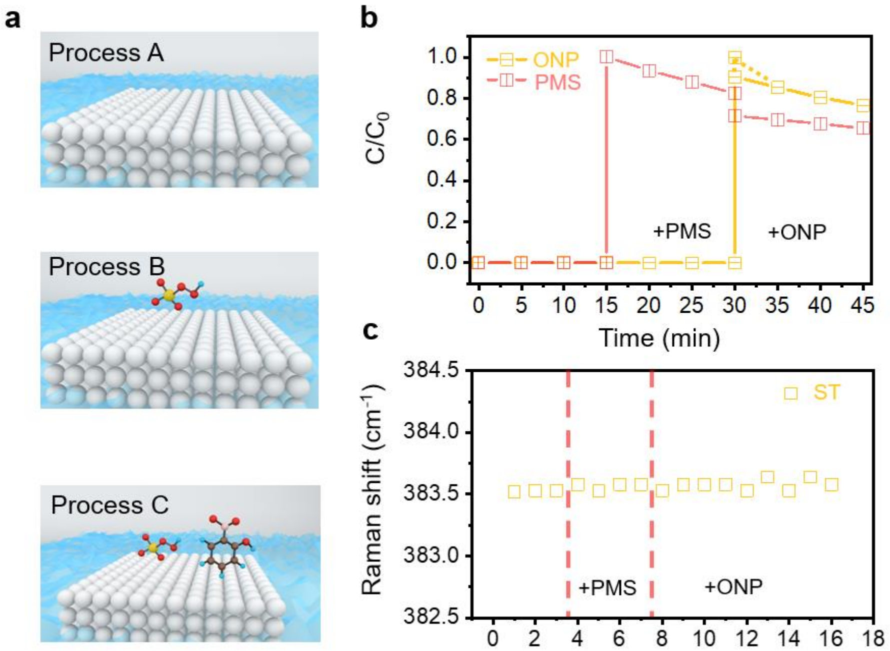

Supplementary Fig. 23. a The reaction sequence of ST of in situ Raman, first add PMS for a period of time and then add ONP. **b** The change of reactant concentration. **c** The change of the Raman shift of peak i.

The Supplementary Fig. 20-23 show two different in situ reaction sequences of ST. During these two in situ reaction processes, there is no obvious change in peak i, Peak ii and Peak iii, even if the concentrations of PMS and ONP are changing, which indicates that ST has not undergone a dynamic change in structure during the reaction.

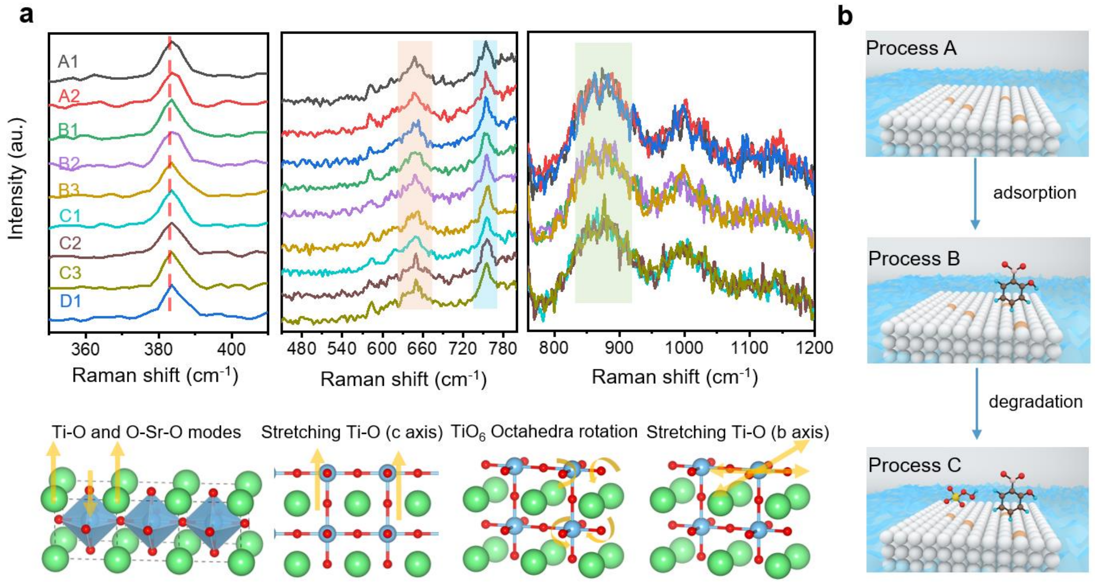

Supplementary Fig. 24. a In situ Raman spectra of STC at different reaction stages, corresponding to **b**. **b** The reaction sequence of in situ Raman, first add ONP for a period of time and then add PMS.

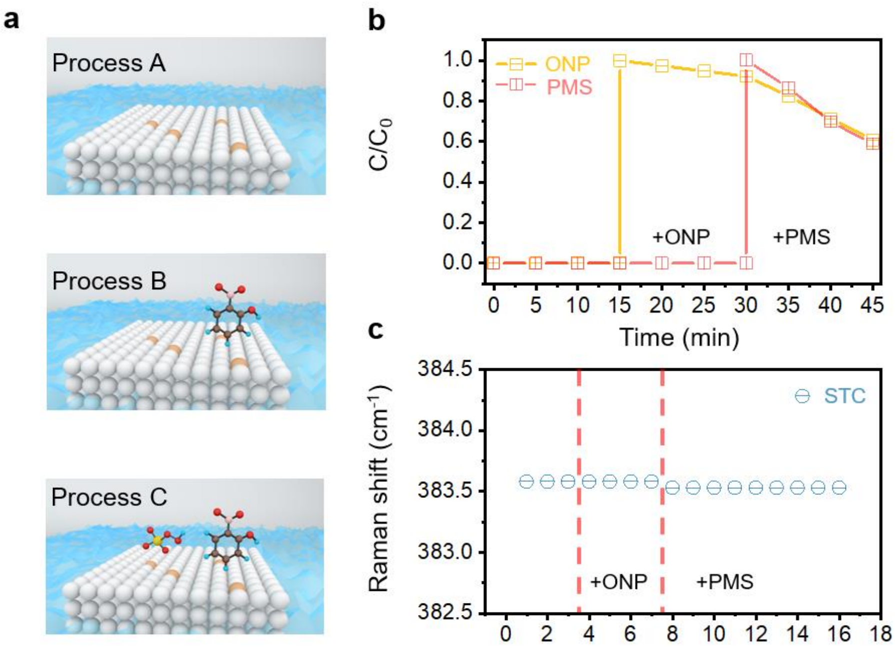

Supplementary Fig. 25. a The reaction sequence of STC of in situ Raman, first add ONP for a period of time and then add PMS. **b** The change of reactant concentration. **c** The change of the Raman shift of peak i.

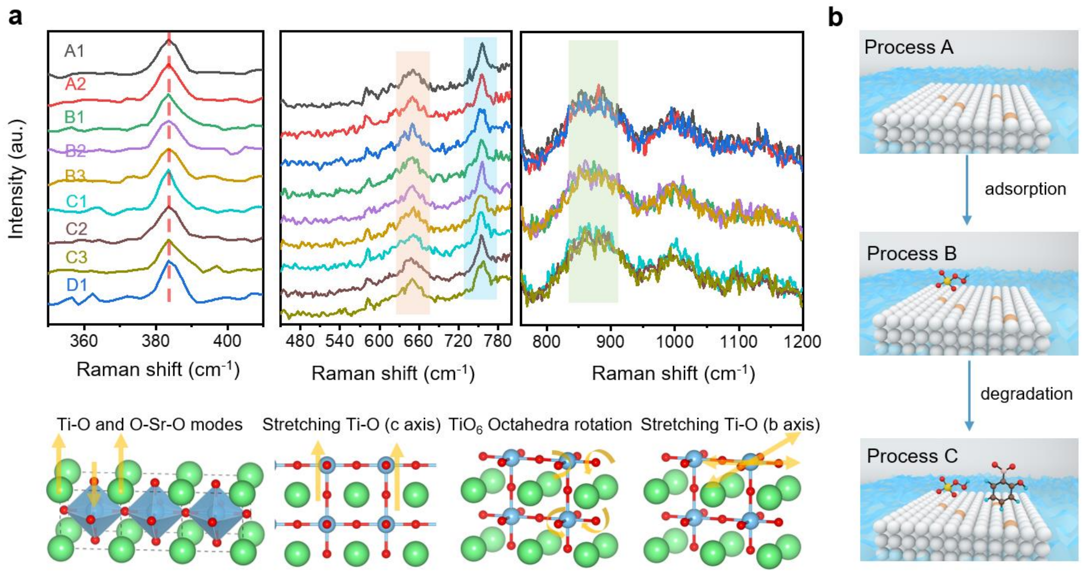

Supplementary Fig. 26. a In situ Raman spectra of STC at different reaction stages, corresponding to **b**. **b** The reaction sequence of in situ Raman, first add PMS for a period of time and then add ONP.

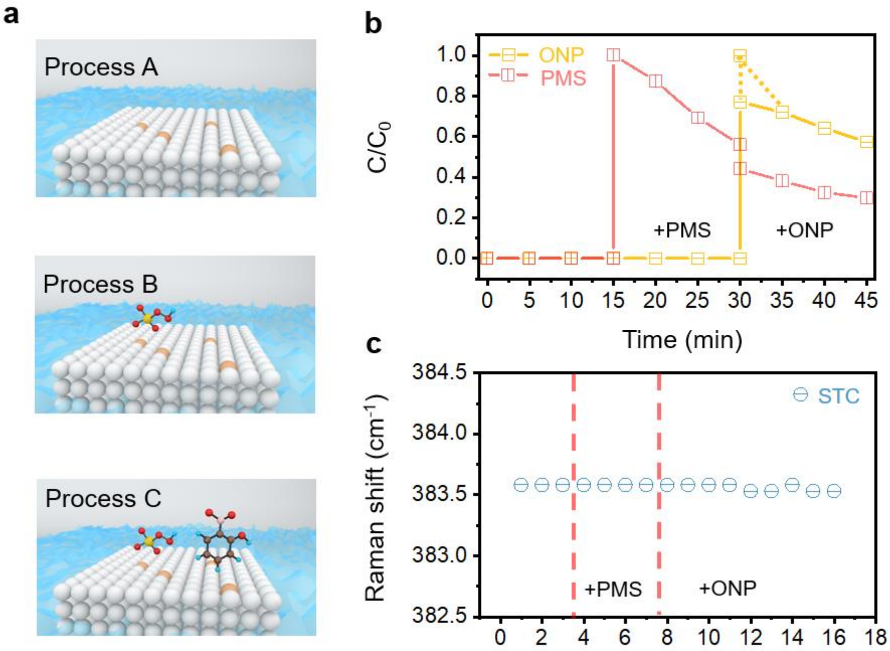

Supplementary Fig. 27. a The reaction sequence of STC of in situ Raman, first add PMS for a period of time and then add ONP. **b** The change of reactant concentration. **c** The change of the Raman shift of peak i.

The Supplementary Figs. 24-27 show two different in situ reaction sequences of STC. During these two in situ reaction processes, there is no obvious change in peak i, Peak ii and Peak iii, even if the concentrations of PMS and ONP are changing, which indicates that STC has not undergone a dynamic change in structure during the reaction.

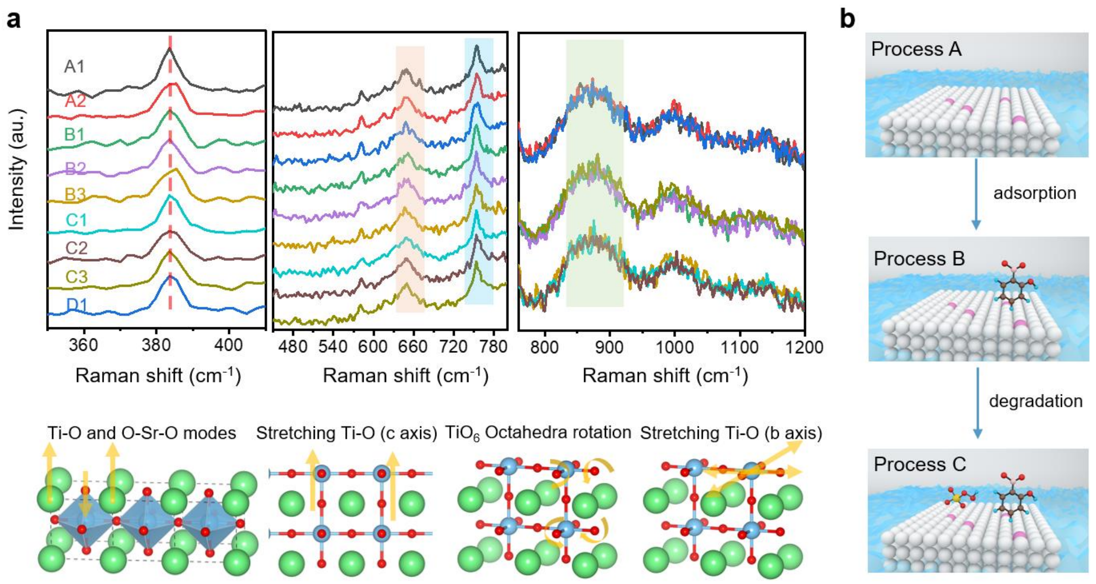

Supplementary Fig. 28. a In situ Raman spectra of STL at different reaction stages, corresponding to **b**. **b** The reaction sequence of in situ Raman, first add ONP for a period of time and then add PMS.

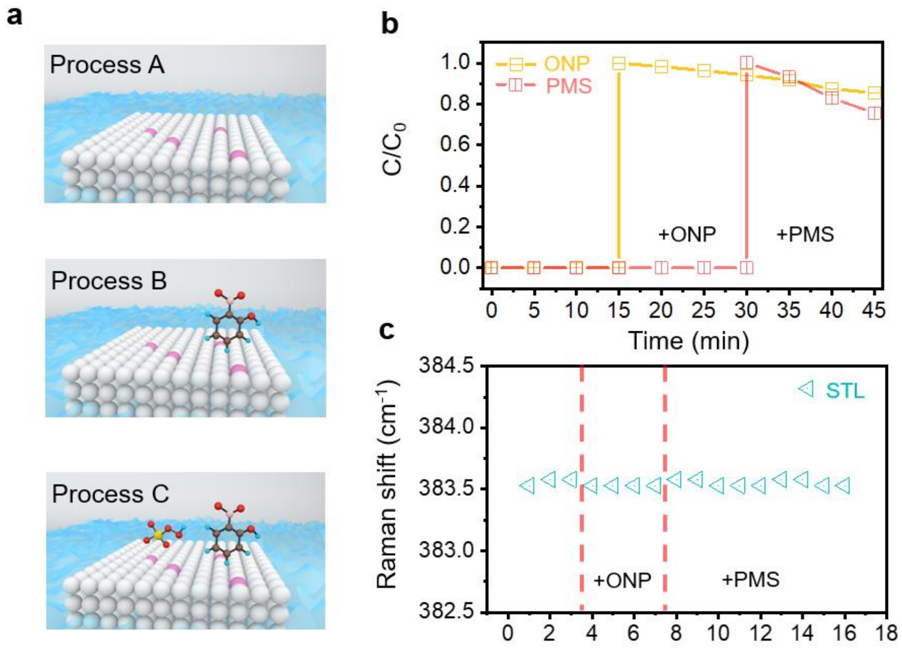

Supplementary Fig. 29. a The reaction sequence of STL of in situ Raman, first add ONP for a period of time and then add PMS. **b** The change of reactant concentration. **c** The change of the Raman shift of peak i.

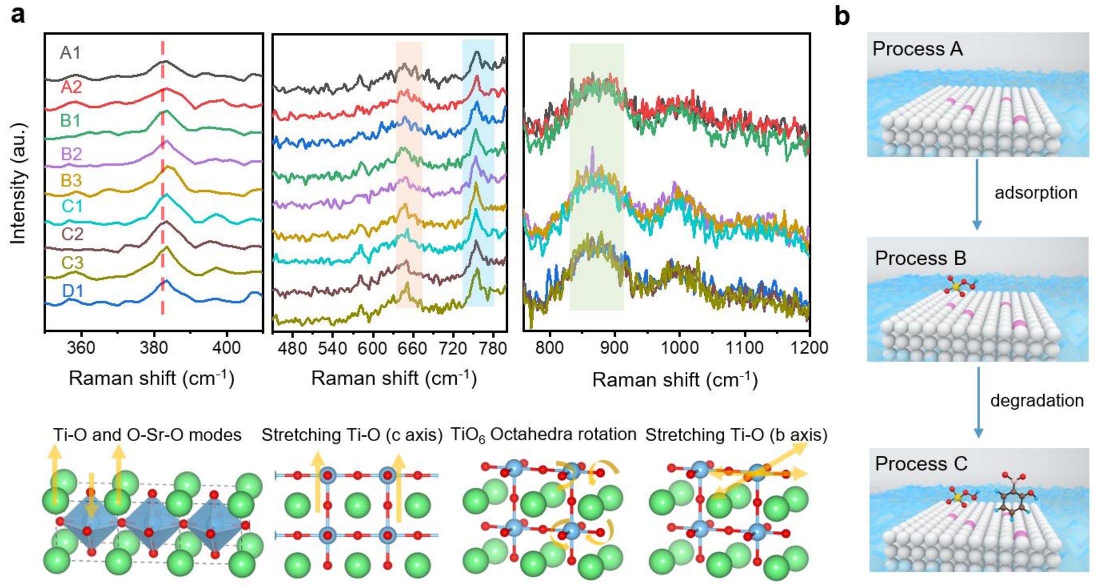

Supplementary Fig. 30. a In situ Raman spectra of STL at different reaction stages, corresponding to **b**. **b** The reaction sequence of in situ Raman, first add PMS for a period of time and then add ONP.

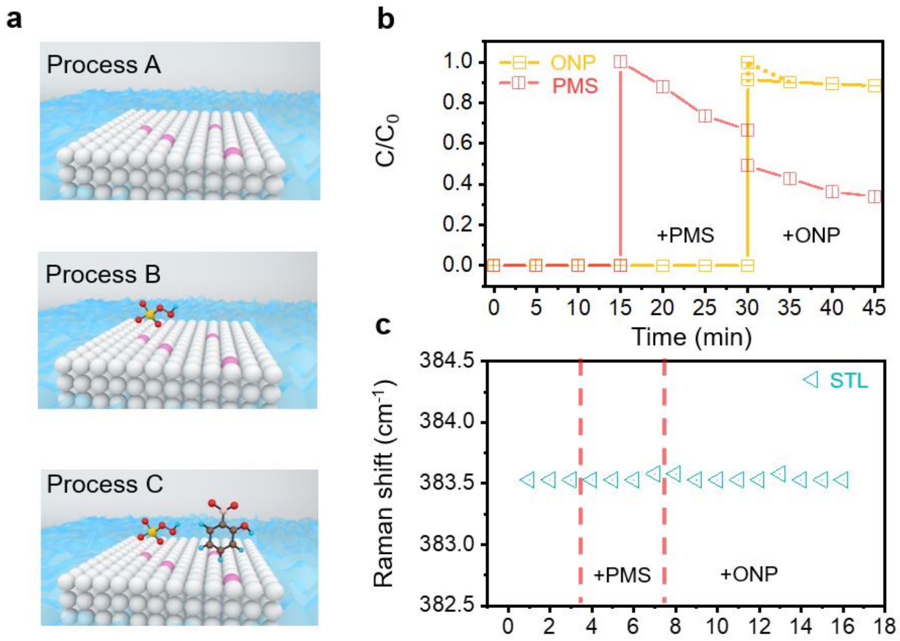

Supplementary Fig. 31. a The reaction sequence of STL of in situ Raman, first add PMS for a period of time and then add ONP. **b** The change of reactant concentration. **c** The change of the Raman shift of peak i.

The Supplementary Figs. 28-31 show two different in situ reaction sequences of STL. During these two in situ reaction processes, there is no obvious change in peak i, Peak ii and Peak iii, even if the concentrations of PMS and ONP are changing, which indicates that STL has not undergone a dynamic change in structure during the reaction.

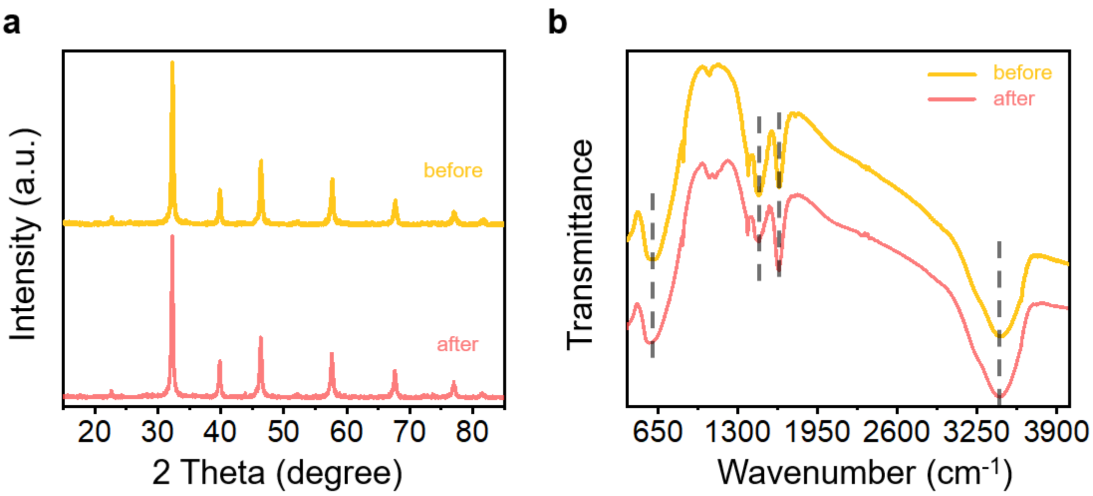

Supplementary Fig. 32. **a** XRD patterns of STLC before and after reaction, **b** The FTIR spectra of STLC before and after reaction. Reaction conditions: initial pollutant 20 mg L^{-1}, initial PMS 0.4 g L^{-1}, catalyst 1.0 g L^{-1}.

These figures show that the structure of the catalyst did not change significantly after the reaction, reflecting the stability of STLC.

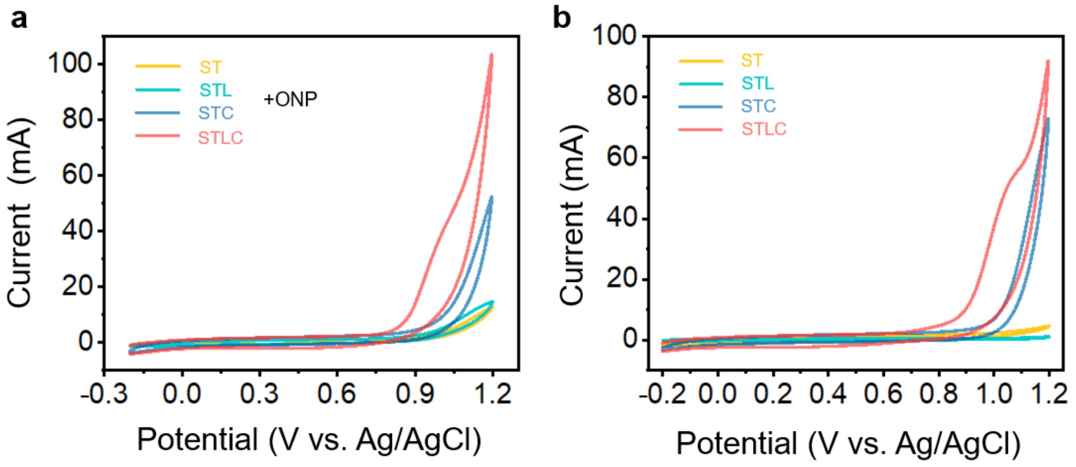

Supplementary Fig. 33. a CV curves on ST, STC, STL and STLC electrode in in 0.1 M Na_2SO_4 solution with 50 ppm ONP. **b** CV curves on ST, STC, STL and STLC electrode in in 0.1 M Na_2SO_4 solution.

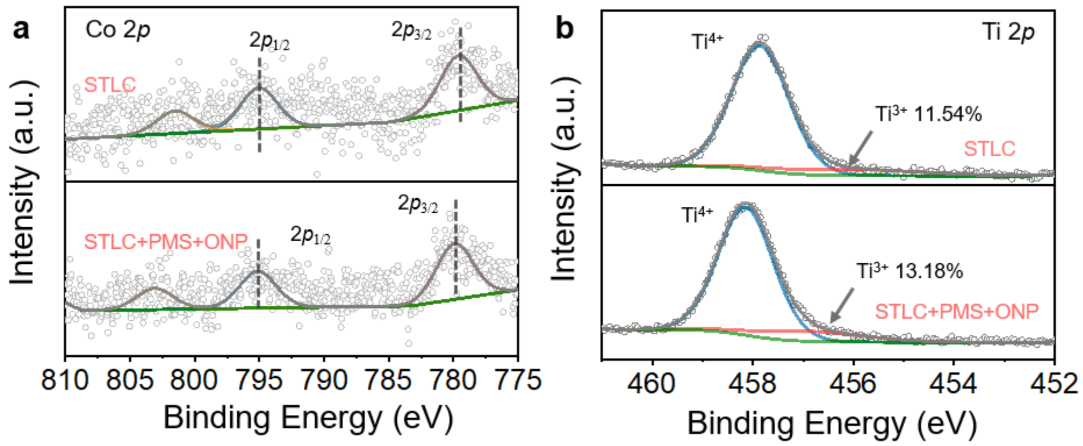

Supplementary Fig. 34. a The Co 2*p* spectra of STLC and during reaction, **b** The Ti 2*p* spectra of STLC before and during reaction.

This figure shows the change of the valence state of Ti during the reaction, Ti^{3+} content changed from 11.54 % to 13.18 %. Reaction conditions: initial pollutant 20 mg L^{-1}, initial PMS 0.4 g L^{-1}, catalyst 1.0 g L^{-1}.

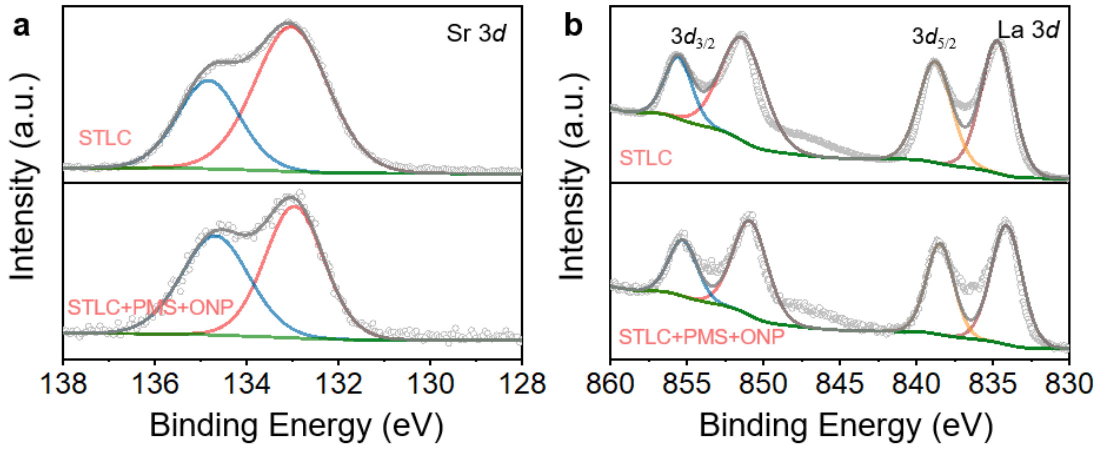

Supplementary Fig. 35. a The Sr 3*d* spectra of STLC and during reaction, **b** The La 3*d* spectra of STLC before and during reaction.

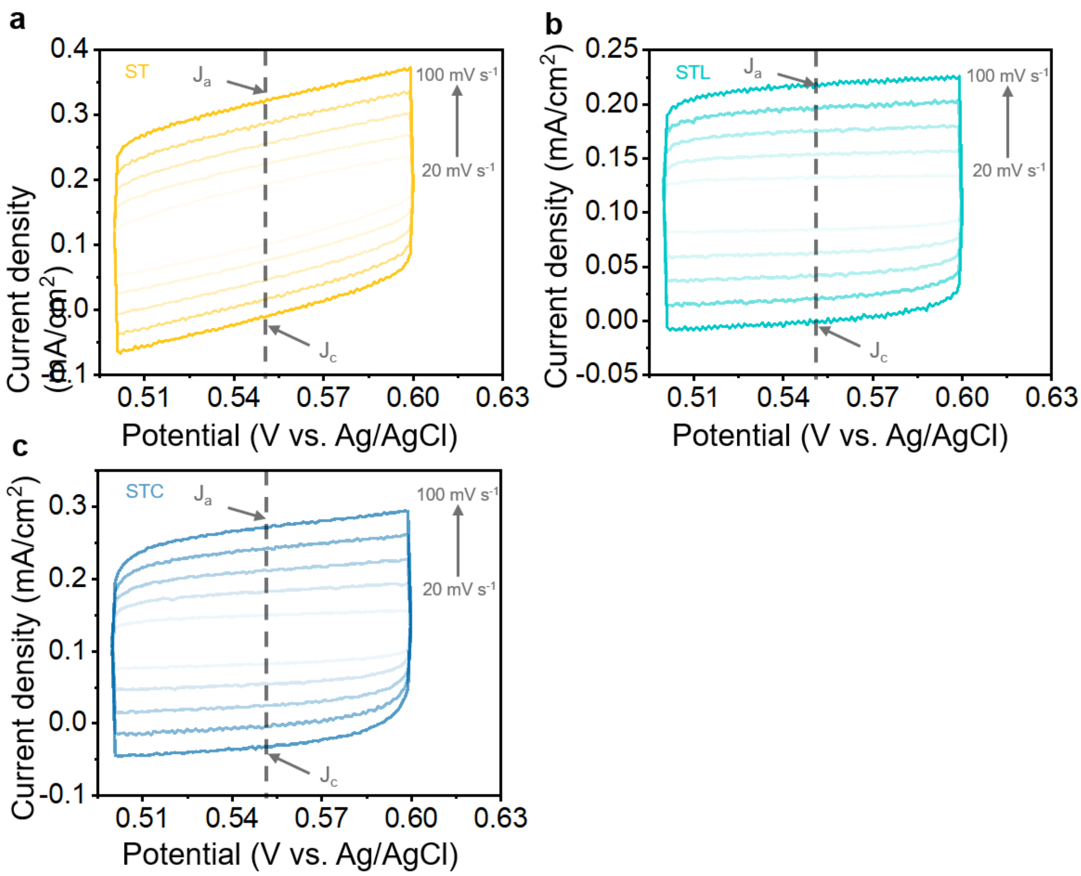

Supplementary Fig. 36. **a** CV curves of at ST different scan rates, **b** CV curves of at STL different scan rates, **c** CV curves of at STC different scan rates.

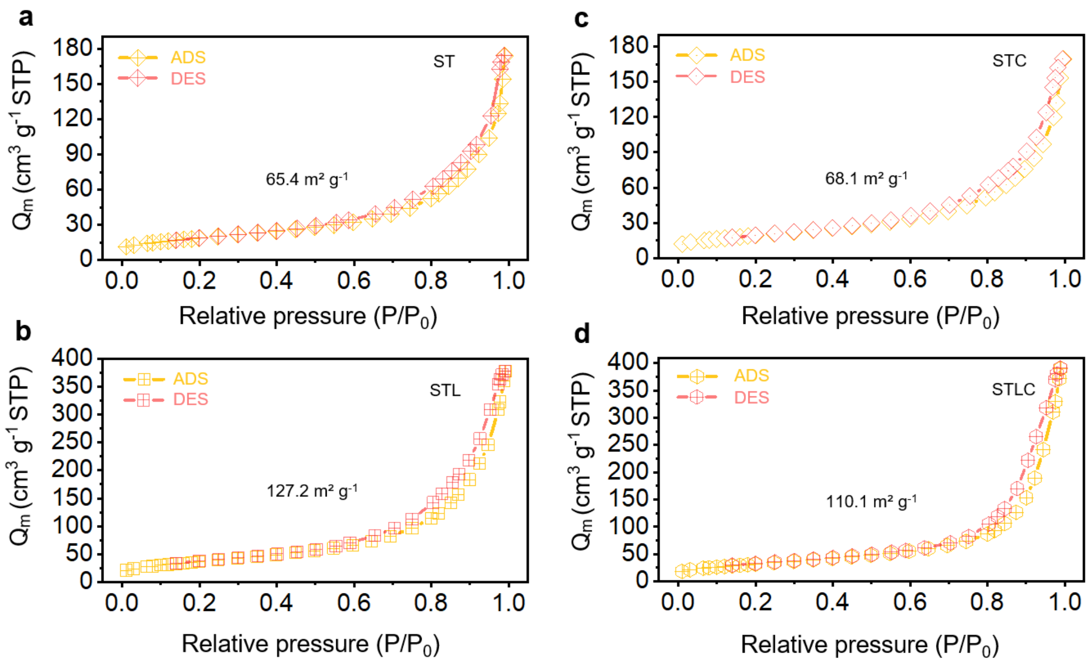

Supplementary Fig. 37. The N_2 adsorption-desorption isotherms of **a** ST; **b** STL; **c** STC; **d** STLC.

The test temperature is 200 °C. As shown in Supplementary Fig. 36, the doping of La increased the specific surface area of the catalyst, but the STL with the highest specific surface area did not show the highest ECSA. Therefore, ECSA is not only affected by the specific surface area.

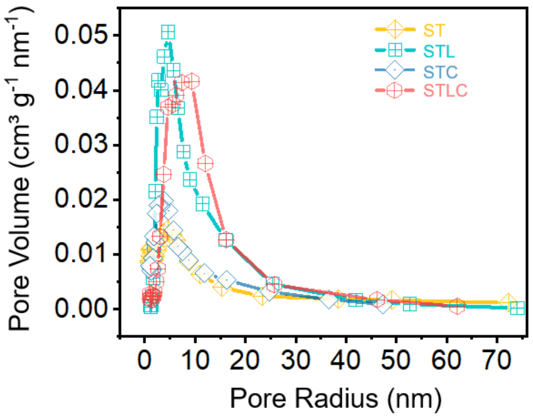

Supplementary Fig. 38. The corresponded pore size distribution of samples.

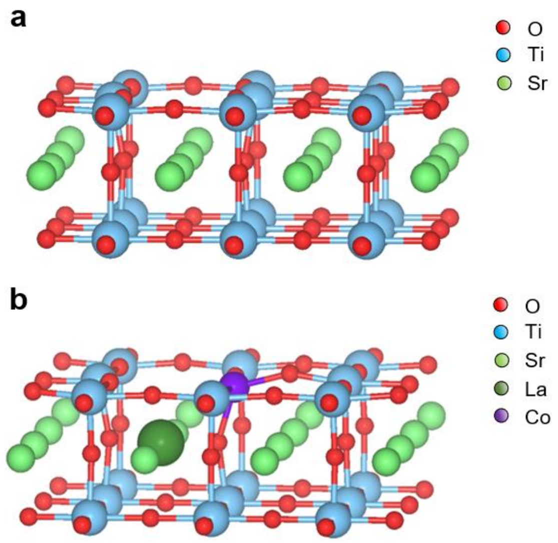

Supplementary Fig. 39. The surface models of ST **a** and STLC **b**.

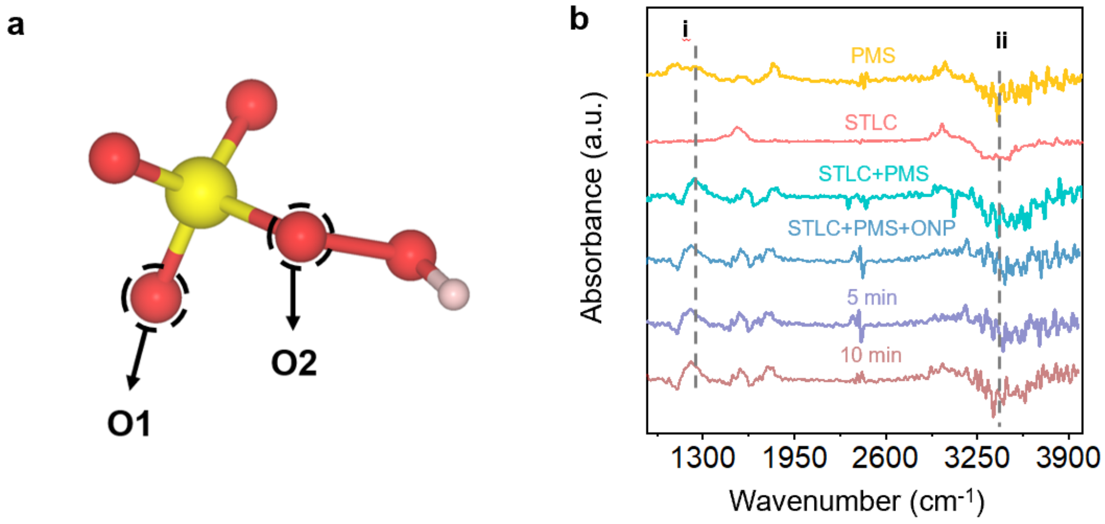

Supplementary Fig. 40. a The O1 and O2 sites on PMS. **b** ATR-FTIR spectra of STLC/PMS/ONP system.

As shown in Fig. 40b, analysis of the catalyst surface by in situ ATR-FTIR revealed a peak at 1241 cm^{-1}, which was the characteristic S-O peaks of HSO_5^-. Furthermore, this peak in the STLC/PMS system exhibited a slight red shift compared to the PMS solution alone, which implied a reduction in the electron density of the S-O bond, causing the HSO_5^- stretching band to become weaker. This result was attributed to the fact that HSO_5^- lost electrons in the surface Co, inhibiting the repulsion of electrons from the neighboring S-O. At the same time, in the process of HSO_5^- losing electrons, SO_5* intermediates were gradually generated ($HSO_5^- \rightarrow SO_5^{\bullet-} + H^+ + e^-$).

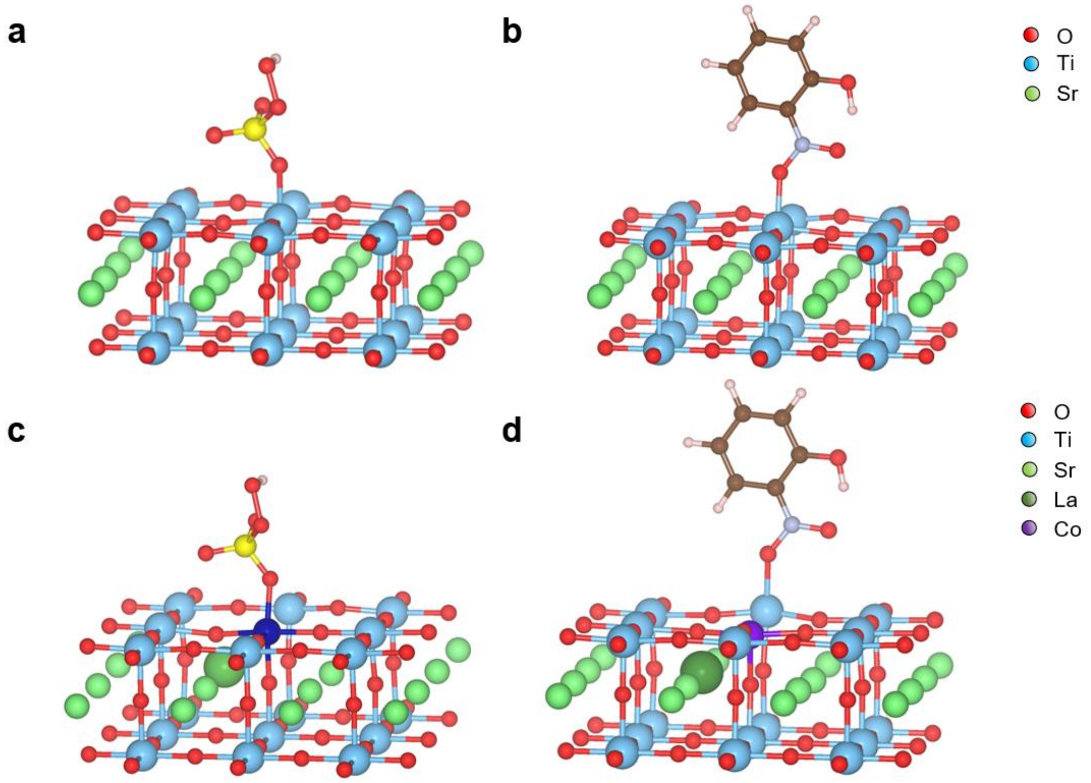

Supplementary Fig. 41. The PMS and ONP were adsorbed on the surface of ST **a**,**b** and STLC **c**,**d** respectively.

The adsorption sites of reaction molecules on ST surface were calculated by DFT, and PMS and ONP tended to be adsorbed on Ti site.

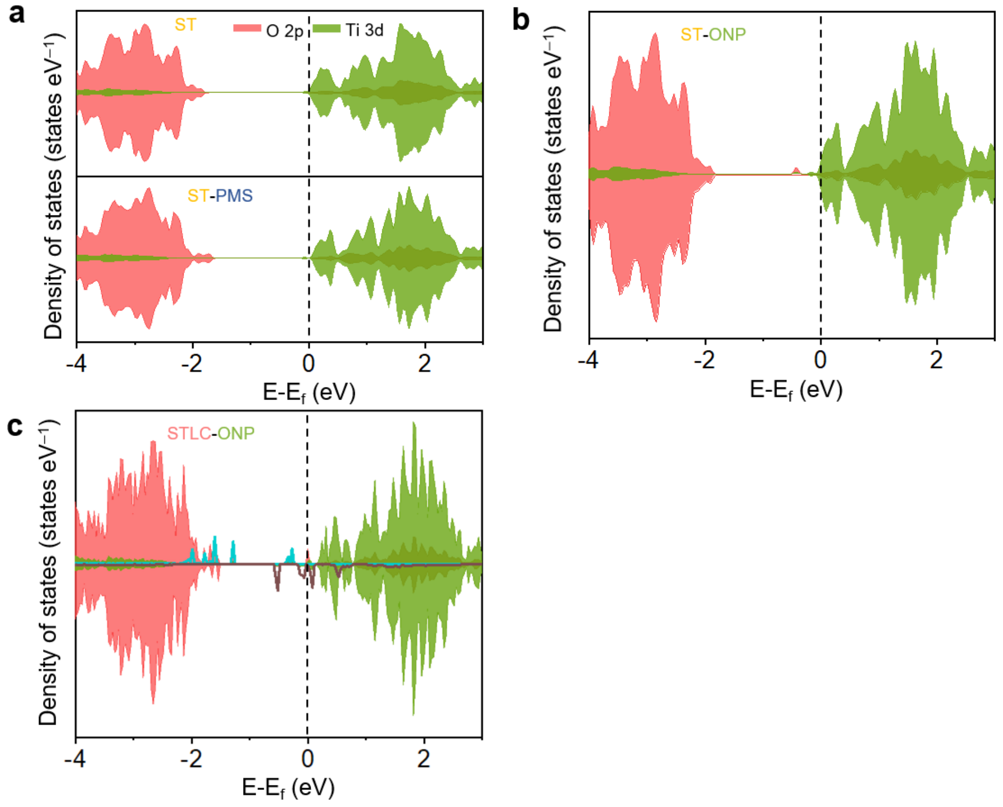

Supplementary Fig. 42. a Projected density of states of ST. Projected density of states of **b** ST and **c** STLC after adsorption of ONP.

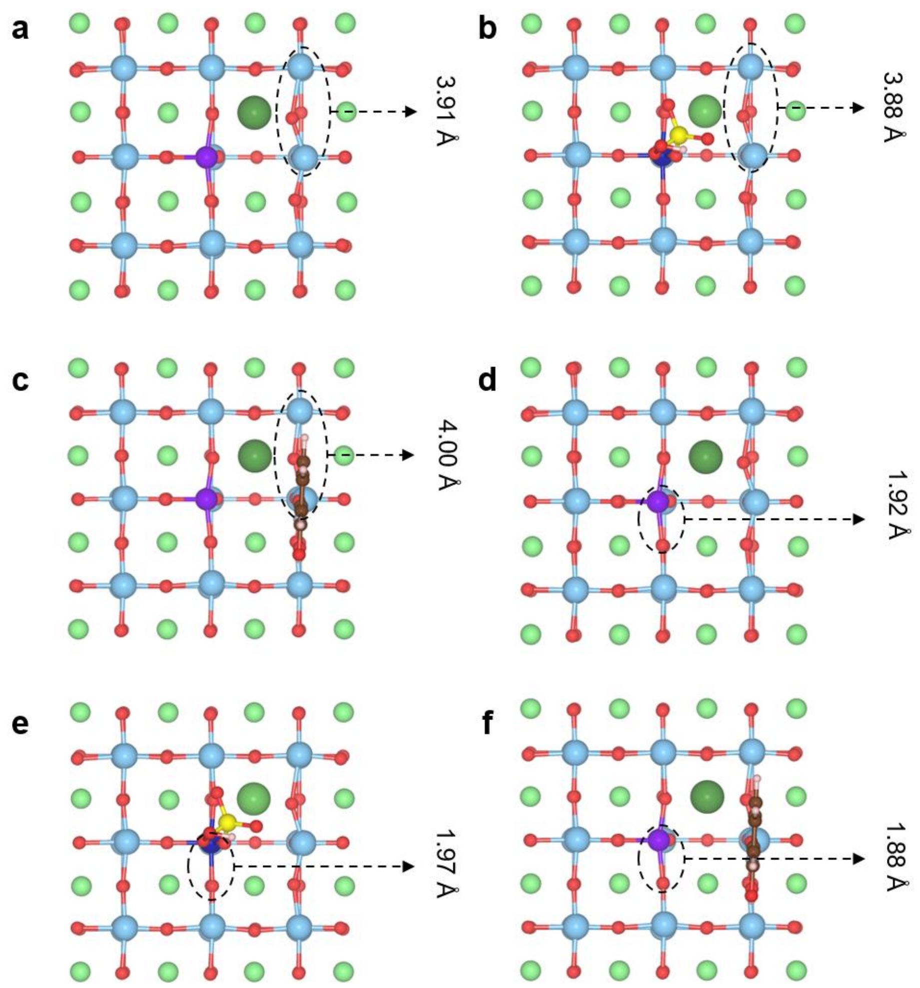

Supplementary Fig. 43. Comparison of bond length before and after adsorption in STLC model. **a**,**b**,**c** The change of bond length of Ti-O-Ti before and after adsorption, **d**,**e**,**f** The change of bond length of Co/Ti-O before and after adsorption. **a**,**d** Bond length of STLC before adsorption, **b**,**e** bond length of STLC after adsorption of PMS, **c**,**f** bond length of STLC after adsorption of ONP.

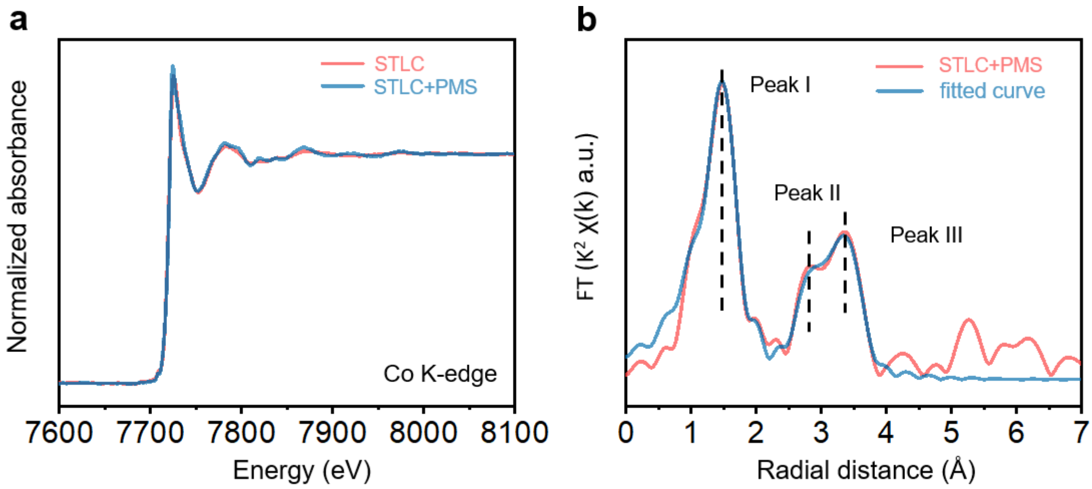

Supplementary Fig. 44. a Normalized Co K-edge XANES spectra of STLC and STLC adsorbed PMS, **b** Co K-edge EXAFS analysis of STLC adsorbed PMS at R space.

Quantitatively, the structural parameters at the Co K-edge were extracted by least-square EXAFS fitting (Supplementary Fig. 44b and Table S1), and the fitting curve matches quite well with the experiment spectra. As indicated by the results, the first shell of the central atom Co displays a coordination number of around 6.8, with mean bond lengths of 1.931 $Å^{-1}$. Compared with STLC, the increased coordination number may be attributed to the binding to O atoms in PMS.

Supplementary Tables

Supplementary Table 1. EXAFS fitting parameters at the Co K-edge for various samples

Sample	Shell	N [a]	R (Å) [b]	σ2 (Å2·10⁻³) [c]	ΔE0 (eV) [d]	R factor (%)
Foil	Co-Co	12*	2.491±0.002	4.21	5.74±0.64	0.2
STC	Co-O	6.1±0.1	1.905±0.017	6.35	7.91±3.37	0.8
STLC	Co-O	5.8±0.2	1.946±0.027	7.82	0.92±5.73	0.4

[a] *N*: coordination numbers; [b] *R*: bond distance; [c] σ^2: Debye-Waller factors; [d] ΔE_0: the inner potential correction. *R* factor: goodness of fit.

S_0^2 was set as 0.85 for Co data, which was obtained from the experimental EXAFS fit of Co foil reference by fixing CN as the known crystallographic value and was fixed to all the samples.

Supplementary Table 2. The calculated adsorption energies of PMS and ONP molecules on $SrTiO_3$ (001) surface with oxygen-vacancy and La, Co co-doped $SrTiO_3$ (001) surface with oxygen-vacancy.

Adsorption system	Molecule	Adsorption energy ΔE_{ad} (eV)
Ov-$SrTiO_3$	PMS at O-vacancy site	−4.428
	PMS at Ti top site	−4.220
	ONP at O-vacancy site	−1.007
	ONP at Ti top site	−1.033
La, Co-Ov-$SrTiO_3$	PMS at Co top site	−3.436
	PMS at O-vacancy site	−1.952
	PMS at Ti top site	−1.086
	ONP at Co top site	−0.683
	ONP at O-vacancy site	−0.573
	ONP at Ti top site	−0.728

Supplementary Table 3. The amount of charge transferred between PMS or ONP molecule and surface.

System	The amount of charge transferred
PMS on O-vacancy site of ov-$SrTiO_3$	0.267e
ONP on Ti top site of ov-$SrTiO_3$	0.713e
PMS on Co top site of La, Co-ov-$SrTiO_3$	0.833e
ONP on Ti top site of La, Co-ov-$SrTiO_3$	0.595e

Supplementary Notes

Supplementary Note 1

The reaction rate constants after adding TBA and MeOH were denoted as k_1 and k_2, respectively, and the initial rate constant without quenching agent was k_0. The contributions of $SO_4^{\bullet-}$, $^1O_2$ and others were calculated according to Eqs. (1)-(3), respectively. The $SO_4^{\bullet-}$ and $^1O_2$ radicals accounted for 65.96% and 33.0% of the contribution to the Fenton-like reaction, respectively. These results indicated that both free radicals played an important role in the degradation of pollutants.

$$\lambda(SO_4^{\bullet-}) = [(k_1\text{-}k_2)/k_0] \times 100\% \quad (3)$$

$$\lambda(^1O_2) = [(k_0\text{-}k_1)/k_0] \times 100\% \quad (4)$$

$$\lambda(\text{others}) = 1\text{-} \lambda(SO_4^{\bullet-}) \text{ - } \lambda(^1O_2) \quad (5)$$

DFT calculations showed that the Bader charge change (0.77 eV) of divided SO_4* and OH* is smaller than the charge change (1.71 eV) of PMS losing H atoms to generate SO_5*. And STLC tended to adsorb the O1 site of PMS on the Co site, which promoted the oxidation of PMS to $SO_5^{\bullet-}$ by the loss of the H atom. Due to the high reaction rate ($\approx 2 \times 10^8$ $M^{-1}s^{-1}$) and low activation energy (7.4 ± 2.4 kcal mol^{-1}), $SO_5^{\bullet-}$ rapidly self-reaction to generate $S_2O_8^{2-}$, $SO_4^{\bullet-}$, and $^1O_2$ [Eq. (3)-(6)].

$$HSO_5^- \rightarrow SO_5^{\bullet-} + H^+ + e^- \quad (6)$$

$$SO_5^{\bullet-} + SO_5^{\bullet-} \rightarrow S_2O_8^{2-} + {}^1O_2 \quad (7)$$

$$SO_5^{\bullet-} + SO_5^{\bullet-} \rightarrow 2SO_4^{\bullet-} + {}^1O_2 \quad (8)$$

In this process, the formation of key intermediate $SO_5^{\bullet-}$ not only facilitated the formation of $^1O_2$ but also produced a large amount of $SO_4^{\bullet-}$.

Supplementary Note 2

In the in situ Raman characterization, the symmetry changes of chemical bonds can be regulated by strain engineering, lattice mismatch, sample surface engineering, and catalyst reconstruction. Two types of distortions occur in the $SrTiO_3$ lattice: ferroelectric shift and the antiferrodistortion rotation. As shown in Fig. 3c, the second-order Raman modes appearing at ~382 cm^{-1} were detected in the in situ Raman, and these peaks were related to the Co/Ti-O and O-Sr-O modes. The shift of these peaks represents the translation of Sr relative to the TiO_6 octahedron and the deformation of the TiO_6 octahedron. Further, the adsorption of reactant molecules to the catalyst enhanced the surface modes of the catalyst. When ONP was added alone, shift of the STLC Raman surface modes (iv) was detected in the in situ Raman spectra, indicating that the deformation of the TiO_6 octahedron. These results lead us to conclude that both the adsorption of ONP and the activation process of PMS cause the local volume distortion of Co/TiO_6 octahedra in the ABO_3 structure.
